# Supplementary material for: Species-specific detection of Schistosoma japonicum using the ‘SNAILS’ DNA-based biosensor
Source: Commun Biol. 2025 Aug 30;8:1321. doi: 10.1038/s42003-025-08773-7 (PMC12398544; doi:10.1038/s42003-025-08773-7)
Supplement: Supplementary file 1 — Supplementary Information [file 42003_2025_8773_MOESM1_ESM.pdf]

Supplementary Files.

Species-specific detection of *Schistosoma japonicum* using the 'SNAILS' DNA-based biosensor

Alexander J. Webb[1], Qin-Ping Zhao[2], Fiona Allan[3,4], Richard J. R. Kelwick[1], Aidan M. Emery[3,4], Paul S. Freemont[1,5,6*]

[1]Section of Structural and Synthetic Biology, Department of Infectious Disease, Imperial College London, London, UK. [2]Department of Parasitology, School of Basic Medical Sciences, Wuhan University, Wuhan 430071, Hubei Province, People's Republic of China. [3]Natural History Museum, London, UK. [4]London School of Hygiene and Tropical Medicine, London, UK. [5]The London Biofoundry, Imperial College Translation and Innovation Hub, White City Campus, London, UK. [6]UK Dementia Research Institute Care Research and Technology Centre, Imperial College London, Hammersmith Campus, London, UK.

[*]Corresponding author: p.freemont@imperial.ac.uk (PSF).

```
KF279410.1      TGAATGATGGTTCCTTCAATATTTTATATGGAATTGAGTTTGTATTATGGATCTGGTGTT
JQ781211.1      TGAATGATGGTTCCTTCAATATTTTATATGGAATTAAGTTTGTATTATGGATCTGGTGTT
JQ781212.1      TGAATGATGGTTCCTTCAATATTTTATATGGAATTAAGTTTGTATTATGGATCTGGTGTT
JQ781208.1      TGAATGATGGTTCCTTCAATATTTTATATGGAATTAAGTTTGTATTATGGATCTGGTGTT
JQ781209.1      TGAATGATGGTTCCTTCAATATTTTATATGGAATTAAGTTTGTATTATGGATCTGGTGTT
JQ781213.1      TGAATGATGGTTCCTTCAATATTTTATATGGAATTAAGTTTGTATTATGGATCTGGTGTT
JQ781206.1      TGAATGATGGTTCCTTCAATATTTTATATGGAATTAAGTTTGTATTATGGATCTGGTGTT
KU196388.1      TGAATGATGGTTCCTTCAATATTTTATATGGAATTAAGTTTGTATTATGGATCTGGTGTT
KU196379.1      TGAATGATGGTTCCTTCAATATTTTATATGGAATTAAGTTTGTATTATGGATCTGGTGTT
LC733209.1      TGAATGATGGTTCCTTCAATATTTTATATGGAATTAAGTTTGTATTATGGATCTGGTGTT
LC733208.1      TGAATGATGGTTCCTTCAATATTTTATATGGAATTAAGTTTGTATTATGGATCTGGTGTT
KU196355.1      TGAATGATGGTTCCTTCAATATTTTATATGGAATTAAGTTTGTATTATGGATCTGGTGTT
JQ781210.1      TGAATGATGGTTCCTTCAATATTTTATATGGAATTAAGTTTGTATTATGGATCTGGTGTT
JQ781214.1      TGAATGATGGTTCCTTCAATATTTTATATGGAATTAAGTTTGTATTATGGGTCTGGTGTT
KU196305.1      TGAATGATGGTTCCTTCAATATTTTATATGGAATTAAGTTTGTATTATGGATCTGGTGTT
EF635954.1      TGAATGATGGTTCCTTCAATATTTTATATGGAATTGAGTTTGTATTATGGATCTGGTGTT
JQ781215.1      TGAATGATGGTTCCTTCAATATTTTATATGGAATTGAGTTTGTATTATGGATCTGGTGTT
KU196343.1      TGAATGATGGTTCCTTCAATATTTTATATGGAATTAAGTTTGTATTATGGATCTGGTGTT
KU196317.1      TGAATGATGGTTCCTTCAATATTTTATATGGAATTAAGTTTGTATTATGGATCTGGTGTT
HM120846.1      TGAATGATGGTTCCTTCAATATTTTATATGGAATTAAGTTTGTATTATGGATCTGGTGTT
KU196374.1      TGAATGATGGTTCCTTCAATATTTTATATGGAATTAAGTTTGTATTATGGATCTGGTGTT
KU196341.1      TGAATGATGGTTCCTTCAATATTTTATATGGAATTAAGTTTGTATTATGGATCTGGTGTT
KU196321.1      TGAATGATGGTTCCTTCAATATTTTATATGGAATTAAGTTTGTATTATGGATCTGGTGTT
KU196301.1      TGAATGATGGTTCCTTCAATATTTTATATGGAATTAAGTTTGTATTATGGATCTGGTGTT
KU196331.1      TGAATGATGGTTCCTTCAATATTTTATATGGAATTAAGTTTGTATTATGGATCTGGTGTT
KU196322.1      TGAATGATGGTTCCTTCAATATTTTATATGGAATTAAGTTTGTATTATGGATCTGGTGTT
KU196312.1      TGAATGATGGTTCCTTCAATATTTTATATGGAATTAAGTTTGTATTATGGATCTGGTGTT
EU325878.1      TGAATGATGGTTCCTTCAATATTTTATATGGAATTAAGTTTGTATTATGGATCTGGTGTT
KU196395.1      TGAATGATGGTTCCTTCAATATTTTATATGGAATTAAGTTTGTATTATGGATCTGGTGTT
KF279409.1      TGAATGATGGTTCCTTCAATATTTTATATGGAATTAAGTTTGTATTATGGATCTGGTGTT
KF279408.1      TGAATGATGGTTCCTTCAATATTTTATATGGAATTAAGTTTGTATTATGGATCTGGTGTT
KF279407.1      TGAATGATGGTTCCTTCAATATTTTATATGGAATTAAGTTTGTATTATGGATCTGGTGTT
HM120847.1      TGAATGATGGTTCCTTCAATATTTTATATGGAATTAAGTTTGTATTATGGATCTGGTGTT
KR855674.1      TGAATGATGGTTCCTTCAATATTTTATATGGAATTAAGTTTGTATTATGGATCTGGTGTT
KU196417.1      TGAATGATGGTTCCTTCAATATTTTATATGGAATTAAGTTTGTATTATGGATCTGGTGTT
KU196413.1      TGAATGATGGTTCCTTCAATATTTTATATGGAATTAAGTTTGTATTATGGATCTGGTGTT
KU196367.1      TGAATGATGGTTCCTTCAATATTTTATATGGAATTAAGTTTGTATTATGGATCTGGTGTT
HM120848.1      TGAATGATGGTTCCTTCAATATTTTATATGGAATTAAGTTTGTATTATGGATCTGGTGTT
                *********************************** ************** *********

KF279410.1      GGTTGGACCTTTTATCCACCTTTGTCTTCTTTAGCTACTTCTGGTGTTGGTGTAGATTAC
JQ781211.1      GGTTGGACCTTTTATCCACCTTTGTCTTCTTTAGCTACTTCTGGTGTTGGTGTGGATTAC
JQ781212.1      GGTTGGACCTTTTATCCACCTTTGTCTTCTTTAGCTACTTCTGGTGTTGGTGTGGATTAC
JQ781208.1      GGTTGGACCTTTTATCCACCTTTGTCTTCTTTAGCTACTTCTGGTGTTGGTGTGGATTAC
JQ781209.1      GGTTGGACCTTTTATCCACCTTTGTCTTCTTTAGCTACTTCTGGTGTTGGTGTGGATTAC
JQ781213.1      GGTTGGGCCTTTTATCCACCTTTGTCTTCTTTAGCTACTTCTGGTGTTGGTGTGGATTAC
JQ781206.1      GGTTGGACCTTTTATCCACCTTTGTCTTCTTTAGCTACTTCTGGTGTTGGTGTGGATTAC
KU196388.1      GGTTGGACTTTTTATCCACCTTTGTCTTCTTTAGCTACTTCTGGTGTTGGTGTGGATTAC
KU196379.1      GGTTGGACTTTTTATCCACCTTTGTCTTCTTTAGCTACTTCTGGTGTTGGTGTGGATTAC
LC733209.1      GGTTGGACTTTTTATCCACCTTTGTCTTCTTTAGCTACTTCTGGTGTTGGTGTGGATTAC
LC733208.1      GGTTGGACTTTTTATCCACCTTTGTCTTCTTTAGCTACTTCTGGTGTTGGTGTGGATTAC
KU196355.1      GGTTGGACCTTTTATCCACCTTTGTCTTCTTTAGCTACTTCTGGTGTTGGTGTGGATTAC
JQ781210.1      GGTTGGACCTTTTATCCACCTTTGTCTTCTTTAGCTACTTCTGGTGTTGGTGTGGATTAC
JQ781214.1      GGTTGGACCTTTTATCCACCTTTGTCTTCTTTAGCTACTTCTGGTGTTGGTGTGGATTAC
KU196305.1      GGTTGGACCTTTTATCCACCTTTGTCTTCTTTAGCTACTTCTGGTGTTGGTGTGGATTAC
EF635954.1      GGTTGGACCTTTTATCCACCTTTGTCTTCTTTAGCTACTTCTGGTGTTGGTGTAGATTAC
JQ781215.1      GGTTGGACCTTTTATCCACCTTTGTCTTCTTTAGCTACTTCTGGTGTTGGTGTAGATTAC
KU196343.1      GGTTGGACTTTTTATCCACCTTTGTCTTCTTTAGCTACTTCTGGTGTTGGTGTGGATTAC
KU196317.1      GGTTGGACCTTTTATCCACCTTTGTCTTCTTTAGCTACTTCTGGTGTTGGTGTGGATTAC
HM120846.1      GGTTGGACCTTTTATCCACCTTTGTCTTCTTTAGCTACTTCTGGTGTTGGTGTGGATTAC
KU196374.1      GGTTGGACCTTTTATCCACCTTTGTCTTCTTTAGCTACTTCTGGTGTTGGTGTGGATTAC
KU196341.1      GGTTGGACCTTTTATCCACCTTTGTCTTCTTTAGCTACTTCTGGTGTTGGTGTGGATTAC
KU196321.1      GGTTGGACCTTTTATCCACCTTTGTCTTCTTTAGCTACTTCTGGTGTTGGTGTGGATTAC
KU196301.1      GGTTGGACCTTTTATCCACCTTTGTCTTCTTTAGCTACTTCTGGTGTTGGTGTGGATTAC
KU196331.1      GGTTGGACCTTTTATCCACCTTTGTCTTCTTTAGCTACTTCTGGTGTTGGTGTGGATTAC
KU196322.1      GGTTGGACCTTTTATCCACCTTTGTCTTCTTTAGCTACTTCTGGTGTTGGTGTGGATTAC
KU196312.1      GGTTGGACCTTTTATCCACCTTTGTCTTCTTTAGCTACTTCTGGTGTTGGTGTGGATTAC
EU325878.1      GGTTGGACCTTTTATCCACCTTTGTCTTCTTTAGCTACTTCTGGTGTTGGTGTGGATTAC
KU196395.1      GGTTGGACCTTTTATCCACCTTTGTCTTCTTTAGCTACTTCTGGTGTTGGTGTGGATTAC
KF279409.1      GGTTGGACCTTTTATCCACCTTTGTCTTCTTTAGCTACTTCTGGTGTTGGTGTGGATTAC
KF279408.1      GGTTGGACCTTTTATCCACCTTTGTCTTCTTTAGCTACTTCTGGTGTTGGTGTGGATTAC
KF279407.1      GGTTGGACCTTTTATCCACCTTTGTCTTCTTTAGCTACTTCTGGTGTTGGTGTGGATTAC
HM120847.1      GGTTGGACCTTTTATCCACCTTTGTCTTCTTTAGCTACTTCTGGTGTTGGTGTAGATTAC
KR855674.1      GGTTGGACCTTTTATCCACCTTTGTCTTCTTTAGCTACTTCTGGTGTTGGTGTGGATTAC
```

```
KU196417.1      GGTTGGACCTTTTATCCACCTTTGTCTTCTTTAGCTACTTCTGGTGTTGGTGTGGATTAC
KU196413.1      GGTTGGACCTTTTATCCACCTTTGTCTTCTTTAGCTACTTCTGGTGTTGGTGTGGATTAC
KU196367.1      GGTTGGACCTTTTATCCACCTTTGTCTTCTTTAGCTACTTCTGGTGTTGGTGTGGATTAC
HM120848.1      GGTTGGACCTTTTATCCACCTTTGTCTTCTTTAGCTACTTCTGGTGTTGGTGTGGATTAC
                ****** * ******************************************** ******

KF279410.1      TTAATGTTCTCTTTACATCTTGCTGGTGTATCTAGTTTGATTGGTTCTATAAATTTTATT
JQ781211.1      TTAATGTTCTCTTTACATCTTGCTGGTGTATCTAGTTTGATTGGTTCTATAAATTTTATT
JQ781212.1      TTAATGTTCTCTTTACATCTTGCTGGTGTATCTAGTTTGATTGGTTCTATAAATTTTATT
JQ781208.1      TTAATGTTCTCTTTACATCTTGCTGGTGTATCTAGTTTGATTGGTTCTATAAATTTTATT
JQ781209.1      TTAATGTTCTCTTTACATCTTGCTGGTGTATCTAGTTTGATTGGTTCTATAAATTTTATT
JQ781213.1      TTAATGTTCTCTTTACATCTTGCTGGTGTATCTAGTTTGATTGGTTCTATAAATTTTATT
JQ781206.1      TTAATGTTCTCTTTACATCTTGCTGGTGTATCTAGTTTGATTGGTTCTATAAATTTTATT
KU196388.1      TTAATGTTCTCTTTACATCTTGCTGGTGTATCTAGTTTGATTGGTTCTATAAATTTTATT
KU196379.1      TTAATGTTCTCTTTACATCTTGCTGGTGTATCTAGTTTGATTGGTTCTATAAATTTTATT
LC733209.1      TTAATGTTCTCTTTACATCTTGCTGGTGTATCTAGTTTGATTGGTTCTATAAATTTTATT
LC733208.1      TTAATGTTCTCTTTACATCTTGCTGGTGTATCTAGTTTGATTGGTTCTATAAATTTTATT
KU196355.1      TTAATGTTCTCTTTACATCTTGCTGGTGTATCTAGTTTGATTGGTTCTATAAATTTTATT
JQ781210.1      TTAATGTTCTCTTTACATCTTGCTGGTGTATCTAGTTTGATTGGTTCTATAAATTTTATT
JQ781214.1      TTAATGTTCTCTTTACATCTTGCTGGTGTATCTAGTTTGATTGGTTCTATAAATTTTATT
KU196305.1      TTAATGTTCTCTTTACATCTTGCTGGTGTATCTAGTTTGATTGGTTCTATAAATTTTATT
EF635954.1      TTAATGTTCTCTTTACATCTTGCTGGTGTATCTAGTTTGATTGGTTCTATAAATTTTATT
JQ781215.1      TTAATGTTCTCTTTACATCTTGCTGGTGTATCTAGTTTGATTGGTTCTATAAATTTTATT
KU196343.1      TTAATGTTCTCTTTACATCTTGCTGGTGTATCTAGTTTGATTGGTTCTATAAATTTTATT
KU196317.1      TTAATGTTCTCTTTACATCTTGCTGGTGTATCTAGTTTGATTGGTTCTATAAATTTTATT
HM120846.1      TTAATGTTCTCTTTACATCTTGCTGGTGTATCTAGTTTGATTGGTTCTATAAATTTTATT
KU196374.1      TTAATGTTCTCTTTACATCTTGCTGGTGTATCTAGTTTGATTGGTTCTATAAATTTTATT
KU196341.1      TTAATGTTCTCTTTACATCTTGCTGGTGTATCTAGTTTGATTGGTTCTATAAATTTTATT
KU196321.1      TTAATGTTCTCTTTACATCTTGCTGGTGTATCTAGTTTGATTGGTTCTATAAATTTTATT
KU196301.1      TTAATGTTCTCTTTACATCTTGCTGGTGTATCTAGTTTGATTGGTTCTATAAATTTTATT
KU196331.1      TTAATGTTCTCTTTACATCTTGCTGGTGTATCTAGTTTGATTGGTTCTATAAATTTTATT
KU196322.1      TTAATGTTCTCTTTACATCTTGCTGGTGTATCTAGTTTGATTGGTTCTATAAATTTTATT
KU196312.1      TTAATGTTCTCTTTACATCTTGCTGGTGTATCTAGTTTGATTGGTTCTATAAATTTTATT
EU325878.1      TTAATGTTCTCTTTACATCTTGCTGGTGTATCTAGTTTGATTGGTTCTATAAATTTTATT
KU196395.1      TTAATGTTCTCTTTACATCTTGCTGGTGTATCTAGTTTGATTGGTTCTATAAATTTTATT
KF279409.1      TTAATGTTCTCTTTACATCTTGCTGGTGTATCTAGTTTGATTGGTTCTATAAATTTTATT
KF279408.1      TTAATGTTCTCTTTACATCTTGCTGGTGTATCTAGTTTGATTGGTTCTATAAATTTTATT
KF279407.1      TTAATGTTCTCTTTACATCTTGCTGGTGTATCTAGTTTGATTGGTTCTATAAATTTTATT
HM120847.1      TTAATGTTCTCTTTACATCTTGCTGGTGTATCTAGTTTGATTGGTTCTATAAATTTTATT
KR855674.1      TTAATGTTCTCTTTACATCTTGCTGGTGTATCTAGTTTGATTGGTTCTATAAATTTTATT
KU196417.1      TTAATGTTCTCTTTACATCTTGCTGGTGTATCTAGTTTGATTGGTTCTATAAATTTTATT
KU196413.1      TTAATGTTCTCTTTACATCTTGCTGGTGTATCTAGTTTGATTGGTTCTATAAATTTTATT
KU196367.1      TTAATGTTCTCTTTACATCTTGCTGGTGTATCTAGTTTGATTGGTTCTATAAATTTTATT
HM120848.1      TTAATGTTCTCTTTACATCTTGCTGGTGTATCTAGTTTGATTGGTTCTATAAATTTTATT
                ************************************************************

KF279410.1      ACTACTATAATGTTGCGTCTAAGGTCATGTTCTTCAGTTATTAGATGATCTTATTTATTT
JQ781211.1      ACTACTATAATGTTGCGTCTAAGGTCATGCTCTTCAGTTATTAGATGATCTTATTTATTT
JQ781212.1      ACTACTATAATGTTGCGTCTAAGGTCATGTTCTTCAGTTATTAGATGATCTTATTTATTT
JQ781208.1      ACTACTATAATGTTGCGTCTAAGGTCATGTTCTTCAGTTATTAGATGATCTTATTTATTT
JQ781209.1      ACTACTATAATGTTGCGTCTAAGGTCATGTTCTTCAGTTATTAGATGATCTTATTTATTT
JQ781213.1      ACTACTATAATGTTGCGTCTAAGGTCATGTTCTTCAGTTATTAGATGATCTTATTTATTT
JQ781206.1      ACTACTATAATGTTGCGTCTAAGGTCATGTTCTTCAGTTATTAGATGATCTTATTTATTT
KU196388.1      ACTACTATAATGTTGCGTCTAAGGTCATGTTCTTCAGTTATTAGATGATCTTATTTATTT
KU196379.1      ACTACTATAATGTTGCGTCTAAGGTCATGTTCTTCAGTTATTAGATGATCTTATTTATTT
LC733209.1      ACTACTATAATGTTGCGTCTAAGGTCATGTTCTTCAGTTATTAGATGATCTTATTTATTT
LC733208.1      ACTACTATAATGTTGCGTCTAAGGTCATGTTCTTCAGTTATTAGATGATCTTATTTATTT
KU196355.1      ACTACTATAATGTTGCGTCTAAGGTCATGTTCTTCAGTTATTAGATGATCTTATTTATTT
JQ781210.1      ACTACTATAATGTTGCGTCTAAGGTCATGTTCTTCAGTTATTAGATGATCTTATTTATTT
JQ781214.1      ACTACTATAATGTTGCGTCTAAGGTCATGTTCTTCAGTTATTAGATGATCTTATTTATTT
KU196305.1      ACTACTATAATGTTGCGTCTAAGGTCATGTTCTTCAGTTATTAGATGATCTTATTTATTT
EF635954.1      ACTACTATAATGTTGCGTCTAAGGTCATGTTCTTCAGTTATTAGATGATCTTATTTATTT
JQ781215.1      ACTACTATAATGTTGCGTCTAAGGTCATGTTCTTCAGTTATTAGATGATCTTATTTATTT
KU196343.1      ACTACTATAATGTTGCGTCTAAGGTCATGTTCTTCAGTTATTAGATGATCTTATTTATTT
KU196317.1      ACTACTATAATGTTGCGTCTAAGGTCATGTTCTTCAGTTATTAGATGATCTTATTTATTT
HM120846.1      ACTACTATAATGTTGCGTCTAAGGTCATGTTCTTCAGTTATTAGATGATCTTATTTATTT
KU196374.1      ACTACTATAATGTTGCGTCTAAGGTCATGTTCTTCAGTTATTAGATGATCTTATTTATTT
KU196341.1      ACTACTATAATGTTGCGTCTAAGGTCATGTTCTTCAGTTATTAGATGATCTTATTTATTT
KU196321.1      ACTACTATAATGTTGCGTCTAAGGTCATGTTCTTCAGTTATTAGATGATCTTATTTATTT
KU196301.1      ACTACTATAATGTTGCGTCTAAGGTCATGTTCTTCAGTTATTAGATGATCTTATTTATTT
KU196331.1      ACTACTATAATGTTGCGTCTAAGGTCATGTTCTTCAGTTATTAGATGATCTTATTTATTT
KU196322.1      ACTACTATAATGTTGCGTCTAAGGTCATGTTCTTCAGTTATTAGATGATCTTATTTATTT
KU196312.1      ACTACTATAATGTTGCGTCTAAGGTCATGTTCTTCAGTTATTAGATGATCTTATTTATTT
EU325878.1      ACTACTATAATGTTGCGTCTAAGGTCATGTTCTTCAGTTATTAGATGATCTTATTTATTT
```

```
KU196395.1      ACTACTATAATGTTGCGTCTAAGGTCATGTTCTTCAGTTATTAGATGATCTTATTTATTT
KF279409.1      ACTACTATAATGTTGCGTCTAAGGTCATGTTCTTCAGTTATTAGATGATCTTATTTATTT
KF279408.1      ACTACTATAATGTTGCGTCTAAGGTCATGTTCTTCAGTTATTAGATGATCTTATTTATTT
KF279407.1      ACTACTATAATGTTGCGTCTAAGGTCATGTTCTTCAGTTATTAGATGATCTTATTTATTT
HM120847.1      ACTACTATAATGTTGCGTCTAAGGTCATGTTCTTCAGTTATTAGATGATCTTATTTATTT
KR855674.1      ACTACTATAATGTTGCGTCTAAGGTCATGTTCTTCAGTTATTAGATGATCTTATTTATTT
KU196417.1      ACTACTATAATGTTGCGTCTAAGGTCATGTTCTTCAGTTATTAGATGATCTTATTTATTT
KU196413.1      ACTACTATAATGTTGCGTCTAAGGTCATGTTCTTCAGTTATTAGATGATCTTATTTATTT
KU196367.1      ACTACTATAATGTTGCGTCTAAGGTCATGTTCTTCAGTTATTAGATGATCTTATTTATTT
HM120848.1      ACTACTATAATGTTGCGTCTAAGGTCATGTTCTTCAGTTATTAGATGATCTTATTTATTT
                ***************************** ******************************

KF279410.1      ACTTCGGTGTTGTTATTGTTATCGTTGCCGGTTCTTGCTGCAGGTATAACTATGTTGTTG
JQ781211.1      ACTTCGGTGTTGTTATTGTTATCGTTGCCGGTTCTTGCTGCAGGTATAACTATGTTGTTG
JQ781212.1      ACTTCGGTGTTGTTATTGTTATCGTTGCCGGTTCTTGCTGCAGGTATAACTATGTTGTTG
JQ781208.1      ACTTCGGTGTTGTTATTGTTATCGTTGCCGGTTCTTGCTGCAGGTATAACTATGTTGTTG
JQ781209.1      ACTTCGGTGTTGTTATTGTTATCGTTGCCGGTTCTTGCTGCAGGTATAACTATGTTGTTG
JQ781213.1      ACTTCGGTGTTGTTATTGTTATCGTTGCCGGTTCTTGCTGCAGGTATAACTATGTTGTTG
JQ781206.1      ACTTCGGTGTTGTTATTGTTATCGTTGCCGGTTCTTGCTGCAGGTATAACTATGTTGTTG
KU196388.1      ACTTCGGTGTTGTTATTGTTATCGTTGCCGGTTCTTGCTGCAGGTATAACTATGTTGTTG
KU196379.1      ACTTCGGTGTTGTTATTGTTATCGTTGCCGGTTCTTGCTGCAGGTATAACTATGTTGTTG
LC733209.1      ACTTCGGTGTTGTTATTGTTATCGTTGCCGGTTCTTGCTGCAGGTATAACTATGTTGTTG
LC733208.1      ACTTCGGTGTTGTTATTGTTATCGTTGCCGGTTCTTGCTGCAGGTATAACTATGTTGTTG
KU196355.1      ACTTCGGTGTTGTTATTGTTATCGTTGCCGGTTCTTGCTGCAGGTATAACTATGTTGTTG
JQ781210.1      ACTTCGGTGTTGTTATTGTTATCGTTGCCGGTTCTTGCTGCAGGTATAACTATGTTGTTG
JQ781214.1      ACTTCGGTGTTGTTATTGTTATCGTTGCCGGTTCTTGCTGCAGGTATAACTATGTTGTTG
KU196305.1      ACTTCGGTGTTGTTATTGTTATCGTTGCCGGTTCTTGCTGCAGGTATAACTATGTTGTTG
EF635954.1      ACTTCGGTGTTGTTATTGTTATCGTTGCCGGTTCTTGCTGCAGGTATAACTATGTTGTTG
JQ781215.1      ACTTCGGTGTTGTTATTGTTATCGTTGCCGGTTCTTGCTGCAGGTATAACTATGTTGTTG
KU196343.1      ACTTCGGTGTTGTTATTGTTATCGTTGCCGGTTCTTGCTGCAGGTATAACTATGTTGTTG
KU196317.1      ACTTCGGTGTTGTTATTGTTATCGTTGCCGGTTCTTGCTGCAGGTATAACTATGTTGTTG
HM120846.1      ACTTCGGTGTTGTTATTGTTATCGTTGCCGGTTCTTGCTGCAGGTATAACTATGTTGTTG
KU196374.1      ACTTCGGTGTTGTTATTGTTATCGTTGCCGGTTCTTGCTGCAGGTATAACTATGTTGTTG
KU196341.1      ACTTCGGTGTTGTTATTGTTATCGTTGCCGGTTCTTGCTGCAGGTATAACTATGTTGTTG
KU196321.1      ACTTCGGTGTTGTTATTGTTATCGTTGCCGGTTCTTGCTGCAGGTATAACTATGTTGTTG
KU196301.1      ACTTCGGTGTTGTTATTGTTATCGTTGCCGGTTCTTGCTGCAGGTATAACTATGTTGTTG
KU196331.1      ACTTCGGTGTTGTTATTGTTATCGTTGCCGGTTCTTGCTGCAGGTATAACTATGTTGTTG
KU196322.1      ACTTCGGTGTTGTTATTGTTATCGTTGCCGGTTCTTGCTGCAGGTATAACTATGTTGTTG
KU196312.1      ACTTCGGTGTTGTTATTGTTATCGTTGCCGGTTCTTGCTGCAGGTATAACTATGTTGTTG
EU325878.1      ACTTCGGTGTTGTTATTGTTATCGTTGCCGGTTCTTGCTGCAGGTATAACTATGTTGTTG
KU196395.1      ACTTCGGTGTTGTTATTGTTATCGTTGCCGGTTCTTGCTGCAGGTATAACTATGTTGTTG
KF279409.1      ACTTCGGTGTTGTTATTGTTATCGTTGCCGGTTCTTGCTGCAGGTATAACTATGTTGTTG
KF279408.1      ACTTCGGTGTTGTTATTGTTATCGTTGCCGGTTCTTGCTGCAGGTATAACTATGTTGTTG
KF279407.1      ACTTCGGTGTTGTTATTGTTATCGTTGCCGGTTCTTGCTGCAGGTATAACTATGTTGTTG
HM120847.1      ACTTCGGTGTTGTTATTGTTATCGTTGCCGGTTCTTGCTGCAGGTATAACTATGTTGTTG
KR855674.1      ACTTCGGTGTTGTTATTGTTATCGTTGCCGGTTCTTGCTGCAGGTATAACTATGTTGTTG
KU196417.1      ACTTCGGTGTTGTTATTGTTATCGTTGCCGGTTCTTGCTGCAGGTATAACTATGTTGTTG
KU196413.1      ACTTCGGTGTTGTTATTGTTATCGTTGCCGGTTCTTGCTGCAGGTATAACTATGTTGTTG
KU196367.1      ACTTCGGTGTTGTTATTGTTATCGTTGCCGGTTCTTGCTGCAGGTATAACTATGTTGTTG
HM120848.1      ACTTCGGTGTTGTTATTGTTATCGTTGCCGGTTCTTGCTGCAGGTATAACTATGTTGTTG
                ************************************************************

KF279410.1      TTTGATCGTAAATTTGGTACTGCTTTTTTTGAGCCAGCAGGTGGTGGTGATCCTGTGTTA
JQ781211.1      TTTGATCGTAAATTTGGTACTGCTTTTTTTGAGCCAGCAGGTGGTGGTGATCCTGTGTTA
JQ781212.1      TTTGATCGTAAATTTGGTACTGCTTTTTTTGAGCCAGCAGGTGGTGGTGATCCTGTGTTA
JQ781208.1      TTTGATCGTAAATTTGGTACTGCTTTTTTTGAGCCAGCAGGTGGTGGTGATCCTGTGTTA
JQ781209.1      TTTGATCGTAAATTTGGTACTGCTTTTTTTGAGCCAGCAGGTGGTGGTGATCCTGTGTTA
JQ781213.1      TTTGATCGTAAATTTGGTACTGCTTTTTTTGAGCCAGCAGGTGGTGGTGATCCTGTGTTA
JQ781206.1      TTTGATCGTAAATTTGGTACTGCTTTTTTTGAGCCAGCAGGTGGTGGTGATCCTGTGTTA
KU196388.1      TTTGATCGTAAATTTGGTACTGCTTTTTTTGAGCCAGCAGGTGGTGGTGATCCTGTGTTA
KU196379.1      TTTGATCGTAAATTTGGTACTGCTTTTTTTGAGCCAGCAGGTGGTGGTGATCCTGTGTTA
LC733209.1      TTTGATCGTAAATTTGGTACTGCTTTTTTTGAGCCAGCAGGTGGTGGTGATCCTGTGTTA
LC733208.1      TTTGATCGTAAATTTGGTACTGCTTTTTTTGAGCCAGCAGGTGGTGGTGATCCTGTGTTA
KU196355.1      TTTGATCGTAAATTTGGTACTGCTTTTTTTGAGCCAGCAGGTGGTGGTGATCCTGTGTTA
JQ781210.1      TTTGATCGTAAATTTGGTACTGCTTTTTTTGAGCCAGCAGGTGGTGGTGATCCTGTGTTA
JQ781214.1      TTTGATCGTAAATTTGGTACTGCTTTTTTTGAGCCAGCAGGTGGTGGTGATCCTGTGTTA
KU196305.1      TTTGATCGTAAATTTGGTACTGCTTTTTTTGAGCCAGCAGGTGGTGGTGATCCTGTGTTA
EF635954.1      TTTGATCGTAAATTTGGTACTGCTTTTTTTGAGCCAGCAGGTGGTGGTGATCCTGTGTTA
JQ781215.1      TTTGATCGTAAATTTGGTACTGCTTTTTTTGAGCCAGCAGGTGGTGGTGATCCTGTGTTA
KU196343.1      TTTGATCGTAAATTTGGTACTGCTTTTTTTGAGCCAGCAGGTGGTGGTGATCCTGTGTTA
KU196317.1      TTTGATCGTAAATTTGGTACTGCTTTTTTTGAGCCAGCAGGTGGTGGTGATCCTGTGTTA
HM120846.1      TTTGATCGTAAATTTGGTACTGCTTTTTTTGAGCCAGCAGGTGGTGGTGATCCTGTGTTA
KU196374.1      TTTGATCGTAAATTTGGTACTGCTTTTTTTGAGCCAGCAGGTGGTGGTGATCCTGTGTTA
KU196341.1      TTTGATCGTAAATTTGGTACTGCTTTTTTTGAGCCAGCAGGTGGTGGTGATCCTGTGTTA
```

```
KU196321.1      TTTGATCGTAAATTTGGTACTGCTTTTTTTGAGCCAGCAGGTGGTGGTGATCCTGTGTTA
KU196301.1      TTTGATCGTAAATTTGGTACTGCTTTTTTTGAGCCAGCAGGTGGTGGTGATCCTGTGTTA
KU196331.1      TTTGATCGTAAATTTGGTACTGCTTTTTTTGAGCCAGCAGGTGGTGGTGATCCTGTGTTA
KU196322.1      TTTGATCGTAAATTTGGTACTGCTTTTTTTGAGCCAGCAGGTGGTGGTGATCCTGTGTTA
KU196312.1      TTTGATCGTAAATTTGGTACTGCTTTTTTTGAGCCAGCAGGTGGTGGTGATCCTGTGTTA
EU325878.1      TTTGATCGTAAATTTGGTACTGCTTTTTTTGAGCCAGCAGGTGGTGGTGATCCTGTGTTA
KU196395.1      TTTGATCGTAAATTTGGTACTGCTTTTTTTGAGCCAGCAGGTGGTGGTGATCCTGTGTTA
KF279409.1      TTTGATCGTAAATTTGGTACTGCTTTTTTTGAGCCAGCAGGTGGTGGTGATCCTGTGTTA
KF279408.1      TTTGATCGTAAATTTGGTACTGCTTTTTTTGAGCCAGCAGGTGGTGGTGATCCTGTGTTA
KF279407.1      TTTGATCGTAAATTTGGTACTGCTTTTTTTGAGCCAGCAGGTGGTGGTGATCCTGTGTTA
HM120847.1      TTTGATCGTAAATTTGGTACTGCTTTTTTTGAGCCAGCAGGTGGTGGTGATCCTGTGTTA
KR855674.1      TTTGATCGTAAATTTGGTACTGCTTTTTTTGAGCCAGCAGGTGGTGGTGATCCTGTGTTA
KU196417.1      TTTGATCGTAAATTTGGTACTGCTTTTTTTGAGCCAGCAGGTGGTGGTGATCCTGTGTTA
KU196413.1      TTTGATCGTAAATTTGGTACTGCTTTTTTTGAGCCAGCAGGTGGTGGTGATCCTGTGTTA
KU196367.1      TTTGATCGTAAATTTGGTACTGCTTTTTTTGAGCCAGCAGGTGGTGGTGATCCTGTGTTA
HM120848.1      TTTGATCGTAAATTTGGTACTGCTTTTTTTGAGCCAGCAGGTGGTGGTGATCCTGTGTTA
                ************************************************************

KF279410.1      TTTCAACATTTATTTTGGTTTTTTGGTCACCCAGAAGTATATGTTTTGATATTGCCTGGG
JQ781211.1      TTTCAACATTTATTTTGGTTTTTTGGTCACCCAGAAGTATATGTTTTGATATTGCCTGGA
JQ781212.1      TTTCAACATTTATTTTGGTTTTTTGGTCACCCAGAAGTATATGTTTTGATATTGCCTGGA
JQ781208.1      TTTCAACATTTATTTTGGTTTTTTGGTCACCCAGAAGTATATGTTTTGATATTGCCTGGA
JQ781209.1      TTTCAACATTTATTTTGGTTTTTTGGTCACCCAGAAGTATATGTTTTGATATTGCCTGGA
JQ781213.1      TTTCAACATTTATTTTGGTTTTTTGGTCACCCAGAAGTATATGTTTTGATATTGCCTGGA
JQ781206.1      TTTCAACATTTATTTTGGTTTTTTGGTCACCCAGAAGTATATGTTTTGATATTGCCTGGA
KU196388.1      TTTCAACATTTATTTTGGTTTTTTGGTCACCCAGAAGTATATGTTTTGATATTGCCTGGA
KU196379.1      TTTCAACATTTATTTTGGTTTTTTGGTCACCCAGAAGTATATGTTTTGATATTGCCTGGA
LC733209.1      TTTCAACATTTATTTTGGTTTTTTGGTCACCCAGAAGTATATGTTTTGATATTGCCTGGA
LC733208.1      TTTCAACATTTATTTTGGTTTTTTGGTCACCCAGAAGTATATGTTTTGATATTGCCTGGA
KU196355.1      TTTCAACATTTATTTTGGTTTTTTGGTCACCCAGAAGTATATGTTTTGATATTGCCTGGA
JQ781210.1      TTTCAACATTTATTTTGGTTTTTTGGTCACCCAGAAGTATATGTTTTGATATTGCCTGGA
JQ781214.1      TTTCAACATTTATTTTGGTTTTTTGGTCACCCAGAAGTATATGTTTTGATATTGCCTGGA
KU196305.1      TTTCAACATTTATTTTGGTTTTTTGGTCACCCAGAAGTATATGTTTTGATATTGCCTGGA
EF635954.1      TTTCAACATTTATTTTGGTTTTTTGGTCACCCAGAAGTATATGTTTTGATATTGCCTGGA
JQ781215.1      TTTCAACATTTATTTTGGTTTTTTGGTCACCCAGAAGTATATGTTTTGATATTGCCTGGA
KU196343.1      TTTCAACATTTATTTTGGTTTTTTGGTCACCCAGAAGTATATGTTTTGATATTGCCTGGA
KU196317.1      TTTCAACATTTATTTTGGTTTTTTGGTCACCCAGAAGTATATGTTTTGATATTGCCTGGA
HM120846.1      TTTCAACATTTATTTTGGTTTTTTGGTCACCCAGAAGTATATGTTTTGATATTGCCTGGA
KU196374.1      TTTCAACATTTATTTTGGTTTTTTGGTCACCCAGAAGTATATGTTTTGATATTGCCTGGA
KU196341.1      TTTCAACATTTATTTTGGTTTTTTGGTCACCCAGAAGTATATGTTTTGATATTGCCTGGA
KU196321.1      TTTCAACATTTATTTTGGTTTTTTGGTCACCCAGAAGTATATGTTTTGATATTGCCTGGA
KU196301.1      TTTCAACATTTATTTTGGTTTTTTGGTCACCCAGAAGTATATGTTTTGATATTGCCTGGA
KU196331.1      TTTCAACATTTATTTTGGTTTTTTGGTCACCCAGAAGTATATGTTTTGATATTGCCTGGA
KU196322.1      TTTCAACATTTATTTTGGTTTTTTGGTCACCCAGAAGTATATGTTTTGATATTGCCTGGA
KU196312.1      TTTCAACATTTATTTTGGTTTTTTGGTCACCCAGAAGTATATGTTTTGATATTGCCTGGA
EU325878.1      TTTCAACATTTATTTTGGTTTTTTGGTCACCCAGAAGTATATGTTTTGATATTGCCTGGA
KU196395.1      TTTCAACATTTATTTTGGTTTTTTGGTCACCCAGAAGTATATGTTTTGATATTGCCTGGA
KF279409.1      TTTCAACATTTATTTTGGTTTTTTGGTCACCCAGAAGTATATGTTTTGATATTGCCTGGA
KF279408.1      TTTCAACATTTATTTTGGTTTTTTGGTCACCCAGAAGTATATGTTTTGATATTGCCTGGA
KF279407.1      TTTCAACATTTATTTTGGTTTTTTGGTCACCCAGAAGTATATGTTTTGATATTGCCTGGA
HM120847.1      TTTCAACATTTATTTTGGTTTTTTGGTCACCCAGAAGTATATGTTTTGATATTGCCTGGA
KR855674.1      TTTCAACATTTATTTTGGTTTTTTGGTCACCCAGAAGTATATGTTTTGATATTGCCTGGA
KU196417.1      TTTCAACATTTATTTTGGTTTTTTGGTCACCCAGAAGTATATGTTTTGATATTGCCTGGA
KU196413.1      TTTCAACATTTATTTTGGTTTTTTGGTCACCCAGAAGTATATGTTTTGATATTGCCTGGA
KU196367.1      TTTCAACATTTATTTTGGTTTTTTGGTCACCCAGAAGTATATGTTTTGATATTGCCTGGA
HM120848.1      TTTCAACATTTATTTTGGTTTTTTGGTCACCCAGAAGTATATGTTTTGATATTGCCTGGA
                ***********************************************************

KF279410.1      TTTGGTATAGTTAGTCATATATGTATGTCTTTAAGTAATAATAATTCTTCGTTTGGATAT
JQ781211.1      TTTGGTATAGTAAGTCATATATGTATGTCTTTAAGTAATAATAATTCTTCGTTTGGATAT
JQ781212.1      TTTGGTATAGTAAGTCATATATGTATGTCTTTAAGTAATAATAATTCTTCGTTTGGATAT
JQ781208.1      TTTGGTATAGTAAGTCATATATGTATGTCTTTAAGTAATAATAATTCTTCGTTTGGATAT
JQ781209.1      TTTGGTATAGTAAGTCATATATGTATGTCTTTAAGTAATAATAATTCTTCGTTTGGATAT
JQ781213.1      TTTGGTATAGTAAGTCATATATGTATGTCTTTAAGTAATAATAATTCTTCGTTTGGATAT
JQ781206.1      TTTGGTATAGTAAGTCATATATGTATGTCTTTAAGTAATAATAATTCTTCGTTTGGATAT
KU196388.1      TTTGGTATAGTAAGTCATATATGTATGTCTTTAAGTAATAATAATTCTTCGTTTGGATAT
KU196379.1      TTTGGTATAGTAAGTCATATATGTATGTCTTTAAGTAATAATAATTCTTCGTTTGGATAT
LC733209.1      TTTGGTATAGTAAGTCATATATGTATGTCTTTAAGTAATAATAATTCTTCGTTTGGATAT
LC733208.1      TTTGGTATAGTAAGTCATATATGTATGTCTTTAAGTAATAATAATTCTTCGTTTGGATAT
KU196355.1      TTTGGTATAGTAAGTCATATATGTATGTCTTTAAGTAATAATAATTCTTCGTTTGGATAT
JQ781210.1      TTTGGTATAGTAAGTCATATATGTATGTCTTTAAGTAATAATAATTCTTCGTTTGGATAT
JQ781214.1      TTTGGTATAGTAAGTCATATATGTATGTCTTTAAGTAATAATAATTCTTCGTTTGGATAT
KU196305.1      TTTGGTATAGTAAGTCATATATGTATGTCTTTAAGTAATAATAATTCTTCGTTTGGATAT
EF635954.1      TTTGGTATAGTAAGTCATATATGTATGTCTTTAAGTAATAATAATTCTTCGTTTGGATAT
```

```
JQ781215.1      TTTGGTATAGTAAGTCATATATGTATGTCTTTAAGTAATAATAATTCTTCGTTTGGATAT
KU196343.1      TTTGGTATAGTAAGTCATATATGTATGTCTTTAAGTAATAATAATTCTTCGTTTGGATAT
KU196317.1      TTTGGTATAGTAAGTCATATATGTATGTCTTTAAGTAATAATAATTCTTCGTTTGGATAT
HM120846.1      TTTGGTATAGTAAGTCATATATGTATGTCTTTAAGTAATAATAATTCTTCGTTTGGATAT
KU196374.1      TTTGGTATAGTAAGTCATATATGTATGTCTTTAAGTAATAATAATTCTTCGTTTGGATAT
KU196341.1      TTTGGTATAGTAAGTCATATATGTATGTCTTTAAGTAATAATAATTCTTCGTTTGGATAT
KU196321.1      TTTGGTATAGTAAGTCATATATGTATGTCTTTAAGTAATAATAATTCTTCGTTTGGATAT
KU196301.1      TTTGGTATAGTAAGTCATATATGTATGTCTTTAAGTAATAATAATTCTTCGTTTGGATAT
KU196331.1      TTTGGTATAGTAAGTCATATATGTATGTCTTTAAGTAATAATAATTCTTCGTTTGGATAT
KU196322.1      TTTGGTATAGTAAGTCATATATGTATGTCTTTAAGTAATAATAATTCTTCGTTTGGATAT
KU196312.1      TTTGGTATAGTAAGTCATATATGTATGTCTTTAAGTAATAATAATTCTTCGTTTGGATAT
EU325878.1      TTTGGTATAGTAAGTCATATATGTATGTCTTTAAGTAATAATAATTCTTCGTTTGGATAT
KU196395.1      TTTGGTATAGTAAGTCATATATGTATGTCTTTAAGTAATAATAATTCTTCGTTTGGATAT
KF279409.1      TTTGGTATAGTAAGTCATATATGTATGTCTTTAAGTAATAATAATTCTTCGTTTGGATAT
KF279408.1      TTTGGTATAGTAAGTCATATATGTATGTCTTTAAGTAATAATAATTCTTCGTTTGGATAT
KF279407.1      TTTGGTATAGTAAGTCATATATGTATGTCTTTAAGTAATAATAATTCTTCGTTTGGATAT
HM120847.1      TTTGGTATAGTAAGTCATATATGTATGTCTTTAAGTAATAATAATTCTTCGTTTGGATAT
KR855674.1      TTTGGTATAGTAAGTCATATATGTATGTCTTTAAGTAATAATAATTCTTCGTTTGGATAT
KU196417.1      TTTGGTATAGTAAGTCATATATGTATGTCTTTAAGTAATAATAATTCTTCGTTTGGATAT
KU196413.1      TTTGGTATAGTAAGTCATATATGTATGTCTTTAAGTAATAATAATTCTTCGTTTGGATAT
KU196367.1      TTTGGTATAGTAAGTCATATATGTATGTCTTTAAGTAATAATAATTCTTCGTTTGGATAT
HM120848.1      TTTGGTATAGTAAGTCATATATGTATGTCTTTAAGTAATAATAATTCTTCGTTTGGATAT
                *********** ************************************************

KF279410.1      TATGGGTTAGTTTGTGCTATGGGTTCTATTGTATGTTTGGGGAGAGTTGTTTGGGCTCAT
JQ781211.1      TGTGGGTTAGTTTGTGCTATGGGTTCTATTGTGTGTTTGGGGAGAGTTGTTTGGGCTCAT
JQ781212.1      TATGGGTTAGTTTGTGCTATGGGTTCTATTGTGTGTTTGGGGAGAGTTGTTTGGGCTCAT
JQ781208.1      TATGGGTTAGTTTGTGCTATGGGTTCTATTGTGTGTTTGGGGAGAGTTGTTTGGGCTCAT
JQ781209.1      TATGGGTTAGTTTGTGCTATGGGTTCTATTGTGTGTTTGGGGAGAGTTGTTTGGGCTCAT
JQ781213.1      TATGGGTTAGTTTGTGCTATGGGTTCTATTGTGTGTTTGGGGAGAGTTGTTTGGGCTCAT
JQ781206.1      TATGGGTTAGTTTGTGCTATGGGTTCTATTGTGTGTTTGGGGAGAGTTGTTTGGGCTCAT
KU196388.1      TATGGGTTAGTTTGTGCTATGGGTTCTATTGTGTGTTTGGGGAGAGTTGTTTGGGCTCAT
KU196379.1      TATGGGTTAGTTTGTGCTATGGGTTCTATTGTGTGTTTGGGGAGAGTTGTTTGGGCTCAT
LC733209.1      TATGGGTTAGTTTGTGCTATGGGTTCTATTGTGTGTTTGGGGAGAGTTGTTTGGGCT---
LC733208.1      TATGGGTTAGTTTGTGCTATGGGTTCTATTGTGTGTTTGGGGAGAGTTGTTTGGGCT---
KU196355.1      TATGGGTTAGTTTGTGCTATGGGTTCTATTGTGTGTTTGGGGAGAGTTGTTTGGGCTCAT
JQ781210.1      TATGGGTTAGTTTGTGCTATGGGTTCTATTGTGTGTTTGGGGAGAGTTGTTTGGGCTCAT
JQ781214.1      TATGGGTTAGTTTGTGCTATGGGTTCTATTGTGTGTTTGGGGAGAGTTGTTTGGGCTCAT
KU196305.1      TATGGGTTAGTTTGTGCTATGGGTTCTATTGTGTGTTTGGGGAGAGTTGTTTGGGCTCAT
EF635954.1      TATGGGTTAGTTTGTGCTATGGGTTCTATTG-----------------------------
JQ781215.1      TATGGGTTAGTTTGTGCTATGGGTTCTATTGTGTGTTTGGGGAGAGTTGTTTGGGCTCAT
KU196343.1      TATGGGTTAGTTTGTGCCATGGGTTCTATTGTGTGTTTGGGGAGAGTTGTTTGGGCTCAC
KU196317.1      TATGGGTTAGTTTGTGCTATGGGTTCTATTGTGTGTTTGGGGAGAGTTGTTTGGGCTCAC
HM120846.1      TATGGGTTAGTTTGTGCTATGGGTTCTATTGTGTGTTTGGGGAGAGTTGTTTGGGCTCAC
KU196374.1      TATGGGTTAGTTTGTGCTATGGGTTCTATTGTGTGTTTGGGGAGAGTTGTTTGGGCTCAC
KU196341.1      TATGGGTTAGTTTGTGCTATGGGTTCTATTGTGTGTTTGGGGAGAGTTGTTTGGGCTCAC
KU196321.1      TATGGGTTAGTTTGTGCTATGGGTTCTATTGTGTGTTTGGGGAGAGTTGTTTGGGCTCAC
KU196301.1      TATGGGTTAGTTTGTGCTATGGGTTCTATTGTGTGTTTGGGGAGAGTTGTTTGGGCTCAC
KU196331.1      TATGGGTTAGTTTGTGCTATGGGTTCTATTGTGTGTTTGGGGAGAGTTGTTTGGGCTCAC
KU196322.1      TATGGGTTAGTTTGTGCTATGGGTTCTATTGTGTGTTTGGGGAGAGTTGTTTGGGCTCAC
KU196312.1      TATGGGTTAGTTTGTGCTATGGGTTCTATTGTGTGTTTGGGGAGAGTTGTTTGGGCTCAC
EU325878.1      TATGGGTTAGTTTGTGCTATGGGTTCTATTGTGTGTTTGGGGAGAGTTGTTTGGGCTCAC
KU196395.1      TATGGGTTAGTTTGTGCTATGGGTTCTATTGTGTGTTTGGGGAGAGTTGTTTGGGCTCAC
KF279409.1      TATGGGTTAGTTTGTGCTATGGGTTCTATTGTGTGTTTGGGGAGAGTTGTTTGGGCTCAC
KF279408.1      TATGGGTTAGTTTGTGCTATGGGTTCTATTGTGTGTTTGGGGAGAGTTGTTTGGGCTCAC
KF279407.1      TATGGGTTAGTTTGTGCTATGGGTTCTATTGTGTGTTTGGGGAGAGTTGTTTGGGCTCAC
HM120847.1      TATGGGTTAGTTTGTGCTATGGGTTCTATTGTGTGTTTGGGGAGAGTTGTTTGGGCTCAC
KR855674.1      TATGGGTTAGTTTGTGCTATGGGTTCTATTGTGTGTTTGGGGAGAGTTGTTTGGGCTCAC
KU196417.1      TATGGGTTAGTTTGTGCTATGGGTTCTATTGTGTGTTTGGGGAGAGTTGTTTGGGCTCAC
KU196413.1      TATGGGTTAGTTTGTGCTATGGGTTCTATTGTGTGTTTGGGGAGAGTTGTTTGGGCTCAC
KU196367.1      TATGGGTTAGTTTGTGCTATGGGTTCTATTGTGTGTTTGGGGAGAGTTGTTTGGGCTCAC
HM120848.1      TATGGGTTAGTTTGTGCTATGGGTTCTATTGTGTGTTTGGGGAGAGTTGTTTGGGCTCAC
```

Supplementary Figure 1. MUSCLE alignment of *cox*1 nucleotide sequences from selected isolates of *Schistosoma japonicum*. The nucleotide sequences accessed from GenBank and aligned here are detailed in Supplementary Table 2. The sequences were aligned using MUSCLE [1] with default parameters. Primer binding sites for the amplification of the 446-base target region are highlighted in grey. The 22-base target regions are also highlighted as follows: probe 2 target (yellow), probe 3 target (blue), probe 4 target (red) and probe 5 target (green). Only relevant portion of the MUSCLE alignment is shown.

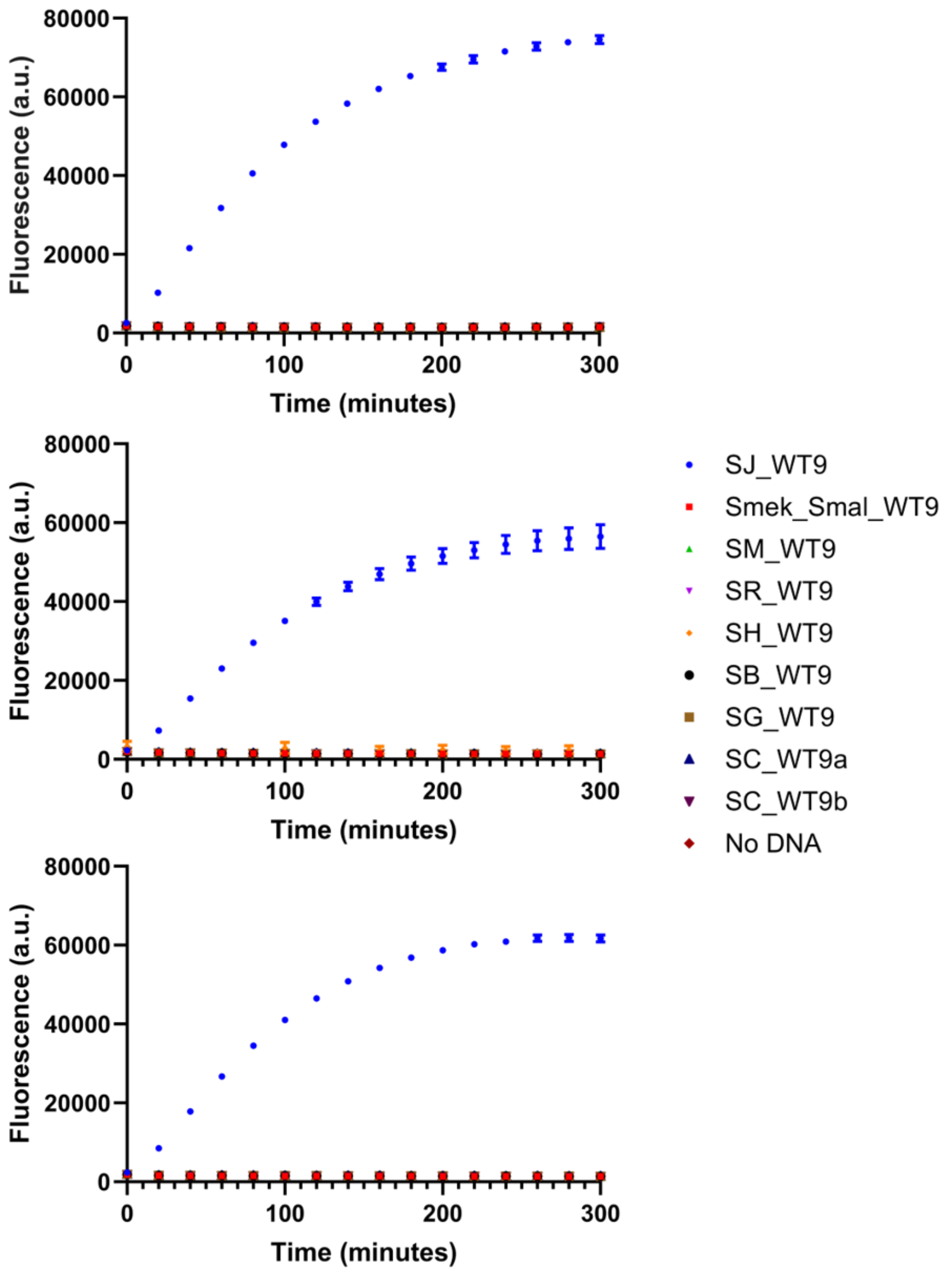

Supplementary Figure 2. Specificity of *S. japonicum* probe set 2 against a range of DNA targets. Both half probes (SJ_A2 and SJ_B2) and the target DNA concentrations were tested at 50 nM. Targets are listed in the key and further details of these targets and the probes are supplied in Supplementary Table 1. Three reaction runs are shown separately, with *n*=3 per graph (1 replicate per reaction, each reaction split into triplicate runs). Measurements were obtained using a BMG CLARIOstar plate reader (Ex. 440-15 nm/ Em. 510-20 nm, 1500 gain). Error bars denote standard error of the mean.

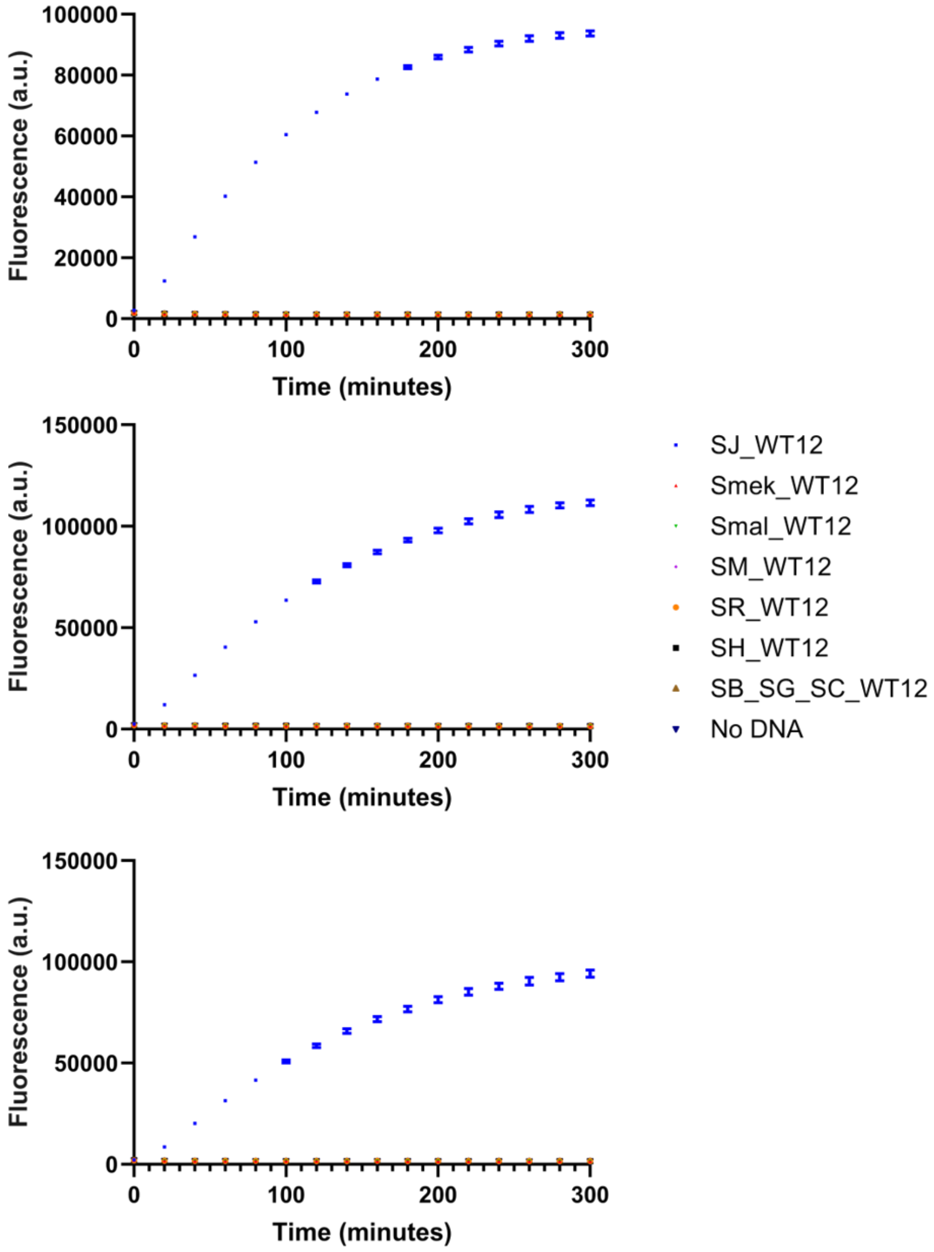

Supplementary Figure 3. Specificity of *S. japonicum* probe set 5 against a range of DNA targets. Both half probes (SJ_A5 and SJ_B5) and the target DNA concentrations were tested at 50 nM. Targets are listed in the key and further details of these targets and the probes are supplied in Supplementary Table 1. Three reaction runs are shown separately, with *n*=3 per graph (1 replicate per reaction, each reaction split into triplicate runs). Measurements were obtained using a BMG CLARIOstar plate reader (Ex. 440-15 nm/ Em. 510-20 nm, 1500 gain). Error bars denote standard error of the mean.

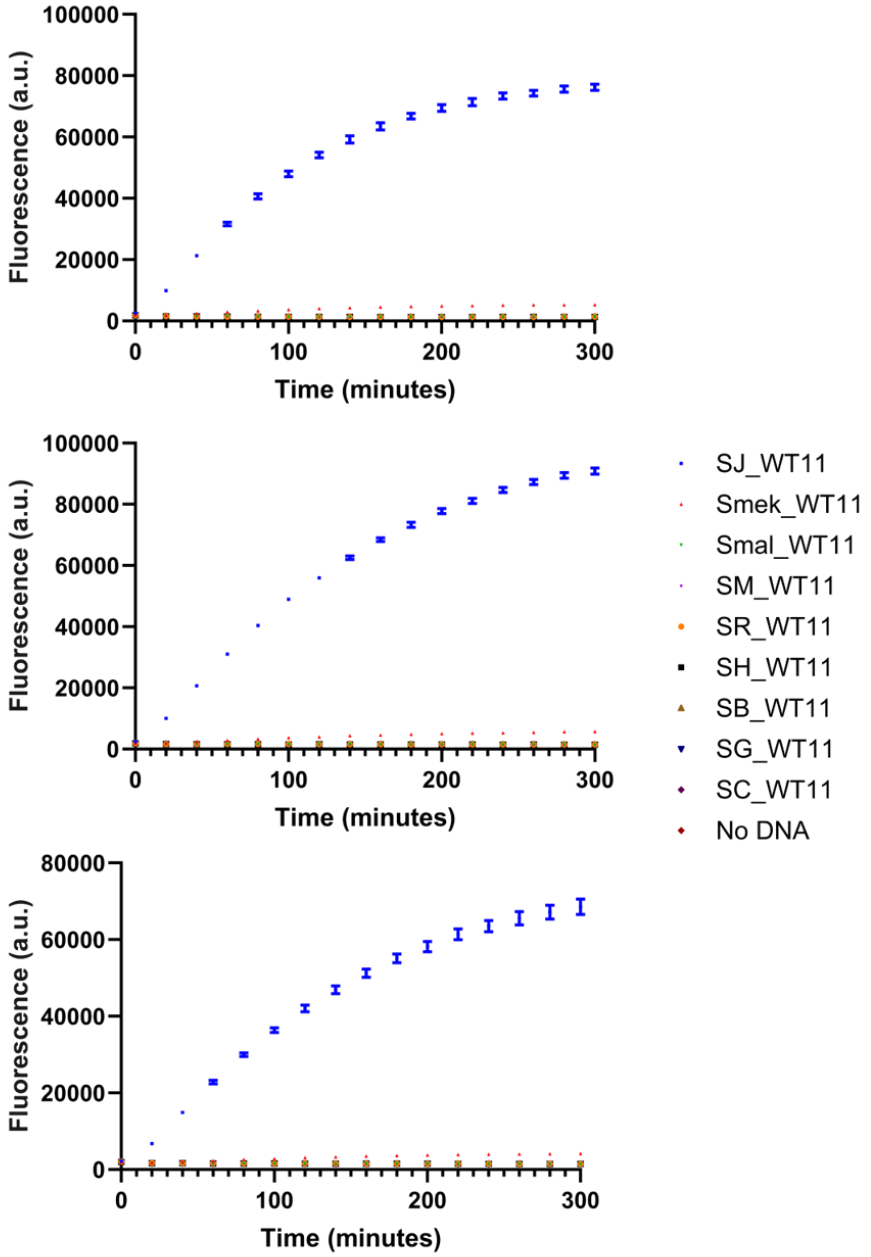

Supplementary Figure 4. Specificity of *S. japonicum* probe set 4 against a range of DNA targets. Both half probes (SJ_A4 and SJ_B4) and the target DNA concentrations were tested at 50 nM. Targets are listed in the key and further details of these targets and the probes are supplied in Supplementary Table 1. Three reaction runs are shown separately, with *n*=3 per graph (1 replicate per reaction, each reaction split into triplicate runs). Measurements were obtained using a BMG CLARIOstar plate reader (Ex. 440-15 nm/ Em. 510-20 nm, 1500 gain). Error bars denote standard error of the mean.

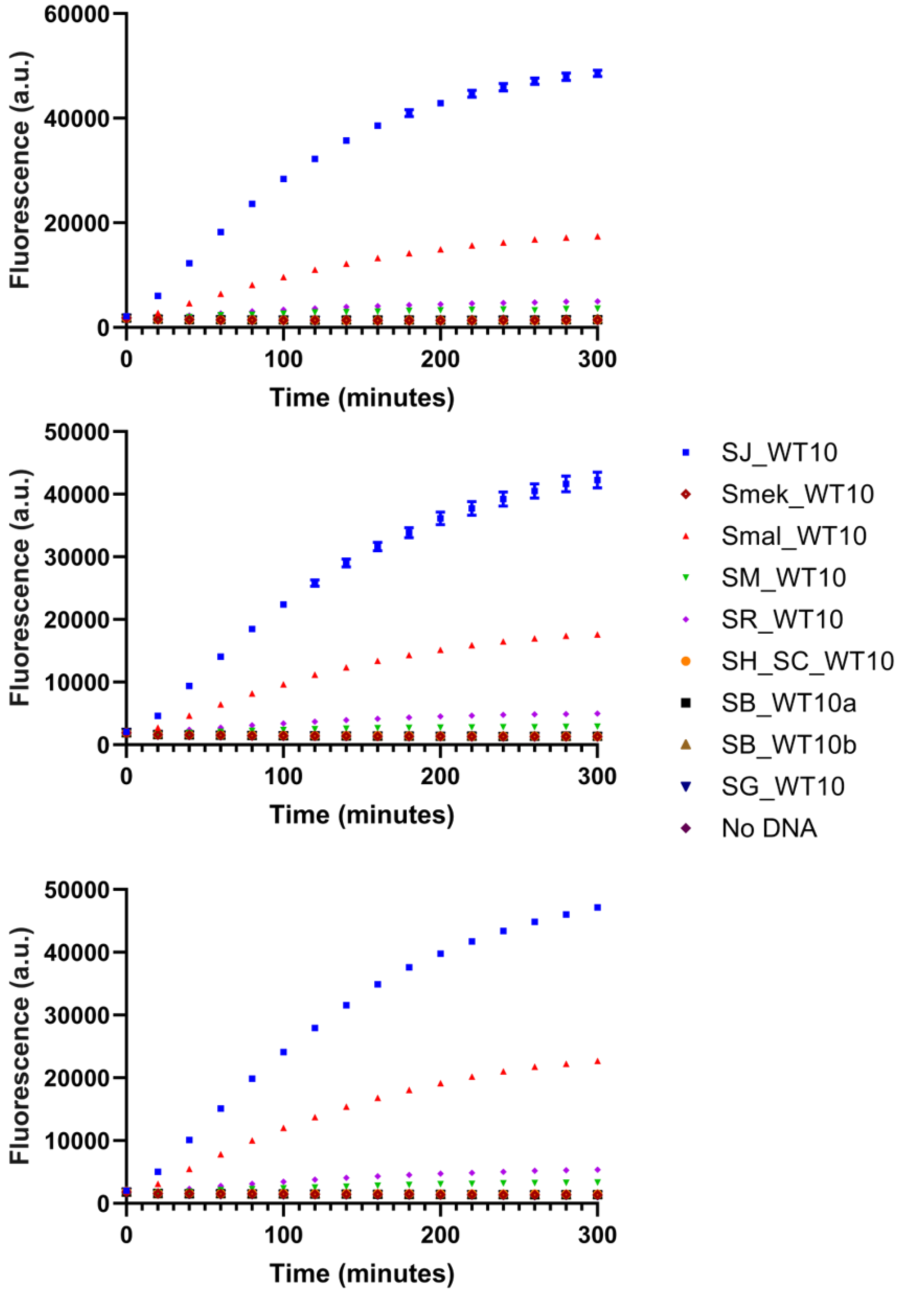

Supplementary Figure 5. Specificity of *S. japonicum* probe set 3 against a range of DNA targets. Both half probes (SJ_A3 and SJ_B3) and the target DNA concentrations were tested at 50 nM. Targets are listed in the key and further details of these targets and the probes are supplied in Supplementary Table 1. Three reaction runs are shown separately, with n=3 per graph (1 replicate per reaction, each reaction split into triplicate runs). Measurements were obtained using a BMG CLARIOstar plate reader (Ex. 440-15 nm/ Em. 510-20 nm, 1500 gain). Error bars denote standard error of the mean.

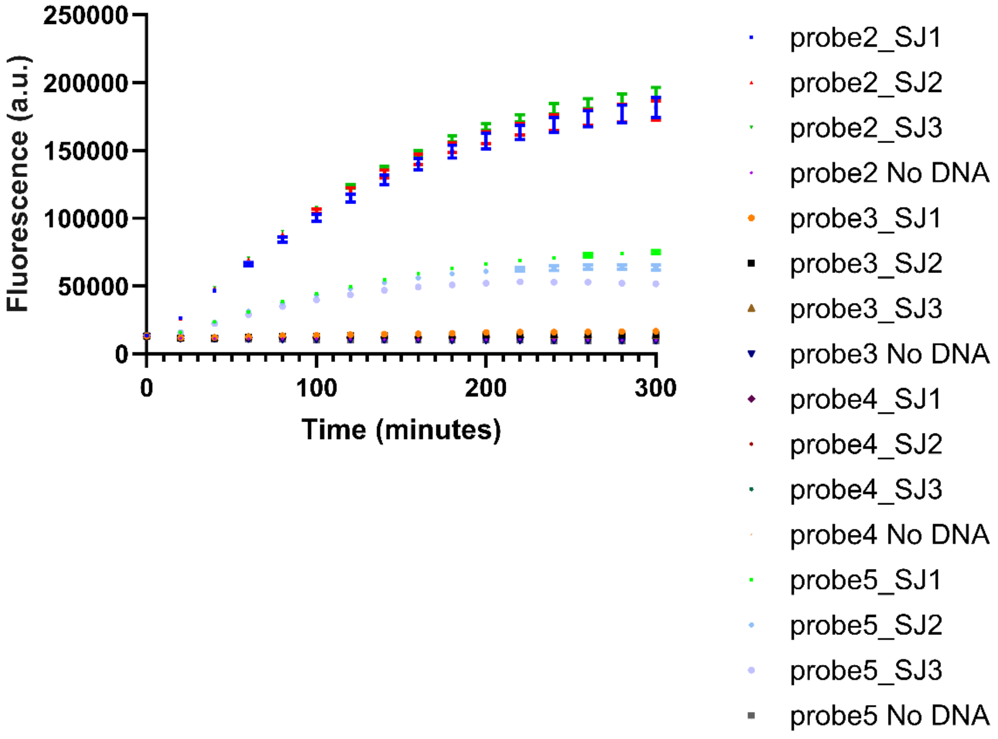

Supplementary Figure 6. Recognition of ssDNA derived from plasmid DNA by *S. japonicum* probe sets 2, 3, 4 and 5. The 446-base target region was PCR amplified from plasmid pAJW333 and treated as described in the main text to produce purified ssDNA. Half probes were tested at 50 nM and the target ssDNA tested at 30 ng. Three PCR reactions were tested (SJ1-SJ3) against each probe set. Reactions are identified as follows: probe2_SJ1-3, probe3_SJ1-3, probe4_SJ1-3 and probe5_SJ1-3 (*S. japonicum* probes 2, 3, 4 and 5 against PCR reactions 1-3 respectively), probe2 No DNA, probe3 No DNA, probe4 No DNA and probe5 No DNA (*S. japonicum* probes 2, 3, 4 and 5 negative controls respectively i.e. no target DNA). n =3 (1 replicate per each reaction split into triplicate runs). Measurements were obtained using a BMG CLARIOstar plate reader (Ex. 440-15 nm/ Em. 510-20 nm, 2000 gain). Error bars denote standard error of the mean.

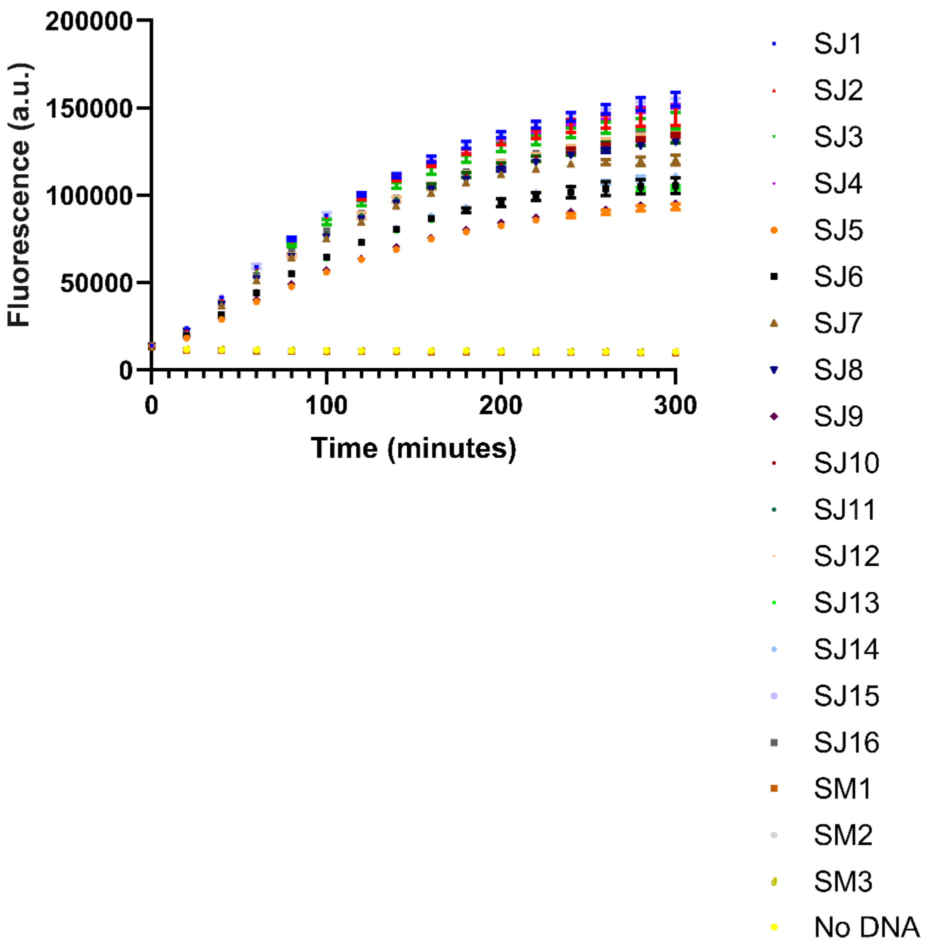

Supplementary Figure 7. Probe 2 detection of ssDNA derived from *S. japonicum* and *S. mansoni* cercariae samples. The 446-base target region was PCR amplified from the *Schistosoma* cercariae samples listed in Supplementary Table 6 and treated as described in the main text to produce purified ssDNA. Probe 2 half probes (SJ_A2 and SJ_B2) were tested at 50 nM and the target ssDNA tested at 30 ng. Reactions are identified as follows: SJ1-SJ16 relate to the ssDNA generated for *S. japonicum* cercariae samples 1-16 tested against probe 2, SM1-SM3 relate to the ssDNA generated for *S. mansoni* cercariae samples 1-3 against probe 2, No DNA is probe 2 negative control (no target ssDNA). *n* =3 (1 replicate per each reaction split into triplicate runs). Measurements were obtained using a BMG CLARIOstar plate reader (Ex. 440-15 nm/ Em. 510-20 nm, 2000 gain). Error bars denote standard error of the mean.

Supplementary Table 1. Oligonucleotide probes and primers used in this study

Number	Name	Sequence (5'-3')
**Probes**		
AJW694	2nd T7 promoter sequence	TAATACGACTCACTATAGGG
AJW1011	SJ_A2	p-ACATTATAGTAGCGATACCCTATAGTGAGTCGTATTA
AJW1012	SJ_B2	GTGTGGGAGCCCACACTCTACTCGACAGATACGAATATCTGGACCCGACCGTCTCCCACACCCTTAGACGCA
AJW1013	SJ_A3	p-ACTTCTGGGTGGCGATACCCTATAGTGAGTCGTATTA
AJW1014	SJ_B3	GTGTGGGAGCCCACACTCTACTCGACAGATACGAATATCTGGACCCGACCGTCTCCCACACTCAAAACATAT
AJW1041	SJ_A4	p-CTAATAACTGAGCGATACCCTATAGTGAGTCGTATTA
AJW1042	SJ_B4	GTGTGGGAGCCCACACTCTACTCGACAGATACGAATATCTGGACCCGACCGTCTCCCACACAATAAGATCAT
AJW1043	SJ_A5	p-CGAAGTAAATAGCGATACCCTATAGTGAGTCGTATTA
AJW1044	SJ_B5	GTGTGGGAGCCCACACTCTACTCGACAGATACGAATATCTGGACCCGACCGTCTCCCACACAATAACAACAC
**Targets**		
AJW1023	SM_WT_9	TACGATTTTTAGTCGTTTAAGA
AJW1024	SR_WT_9	TACTATTCTAAGTCGTTTAAGG
AJW1025	SH_WT_9	TACGATTATTAGTCGTGTCAAT
AJW1026	SB_WT_9	TACAATTATGAGTTGTGTAGAT
AJW1027	SG_WT_9	TACGATTATTAGTTGTGTTGAT
AJW1028	SC_WT_9_AY157210	TACAATTATTAGTCGTGTTGAT
AJW1029	SC_WT_9_AJ519516	TACAATTATCAGTCGTGTTGAT
AJW1030	SJ_WT_9	TACTATAATGTTGCGTCTAAGG
AJW1031	Smek_Smal_WT_9	TACAATAATGATACGTTTAAAT
AJW1032	SM_WT_10	CATCCAGAGGTTTATGTTTTGA
AJW1033	SR_WT_10	CATCCAGAAGTTTATGTTTTAA
AJW1034	SH_SC_WT_10	CATCCGGAGGTGTATGTTTTAA
AJW1035	SB_WT_10_MH647124	CATCCGGAGGTGTATGTTTTGA
AJW1036	SB_WT_10_FJ897160	CATCCTGAGGTGTATGTTTTGA
AJW1037	SG_WT_10	CATCCGGAGGTGTACGTTTTGA
AJW1038	SJ_WT_10	CACCCAGAAGTATATGTTTTGA
AJW1039	Smek_WT_10	CATCCTGAGGTTTATGTTTTAA
AJW1040	Smal_WT_10	CATCCTGAAGTTTATGTTTTGA
AJW1045	SJ_WT_11	TCAGTTATTAGATGATCTTATT
AJW1046	Smek_WT_11	TCAGTTATAAGTTGATCTTATT
AJW1047	Smal_WT_11	TCGGTTATAAGTTGGTCTTATT
AJW1048	SM_WT_11	TCGATAATAGTATGGGCTTATC
AJW1049	SR_WT_11	TCGATAATAGTGTGGGCTTATT
AJW1050	SH_WT_11	TCTATAATAATATGATCATATT
AJW1051	SB_WT_11	TCGATAATAATCTGATCATATT
AJW1052	SG_WT_11	TCAATAATAATTTGGTCATATT
AJW1053	SC_WT_11	TCAATAATAATCTGGTCATATT
AJW1054	SJ_WT_12	TATTTACTTCGGTGTTGTTATT
AJW1055	Smek_WT_12	TTTTTACTTCTATACTTTTGTT
AJW1056	Smal_WT_12	TTTTTACTTCTATACTTTTATT
AJW1057	SM_WT_12	TATTTACGTCTGTCTTACTATT
AJW1058	SR_WT_12	TATTTACATCTATTCTTTTATT
AJW1059	SH_WT_12	TGTTCACTTCTATCTTATTATT
AJW1060	SB_SG_SC_WT_12	TATTTACTTCAATTTTATTGTT
**Primers for target amplification and/or sequencing**		
AJW1061	5-SJ-cox1	CCTTTGTCTTCTTTAGCTACTTCTGG
AJW1062	3-SJ-cox1	CTCCCCAAACACACAATAGAACCCA

AJW1063	p-3-SJ-cox1	p-CTCCCCAAACACACAATAGAACCCA
AJW1064	5-SM-cox1	CCTTTATCAATTTGAGAGGGGTCTGG
AJW1065	3-SM-cox1	CTACCTAAGCATACTATAGAAGCCA
AJW1065	p-3-SM-cox1	p-CTACCTAAGCATACTATAGAAGCCA

The T7 promoter sequence, target sequences and the 'Spinach' aptamer sequence for the probes are indicated by red, blue and green text respectively. Phosphorylated primers are indicated by 'p-'. The 2nd T7 promoter primer sequence (AJW694) was taken from[2,3].

Supplementary Table 2. GenBank accession numbers of the *Schistosoma japonicum cox*1 gene sequences used to valid biosensor targets

Strain/Isolate	Accession number	Location isolated	Reference
Yunnan DaLi (SjYD)	EU325878.1	China: Yunnan province	GenBank
SJLEYM5	JQ781210.1	Philippines: Leyte	GenBank
SJ7_pilon	KR855674.1	China: Dali, Yunnan	[4]
YNEY10	KU196417.1	-	GenBank
YNEY06	KU196413.1	-	GenBank
JXDC09	KU196367.1	-	GenBank
SJYEIIF10	HM120848.1	China: Yunnan, Eryuan	GenBank
SCXC08	KU196395.1	-	GenBank
JXNC06	KU196374.1	-	GenBank
HNYY04	KU196341.1	-	GenBank
HBSS04	KU196321.1	-	GenBank
AHGC03	KU196301.1	-	GenBank
Yunnan Heqing	KF279409.1	China: Yunnan, Heqing	GenBank
Yunnan Eryuan	KF279408.1	China: Yunnan, Eryuan	GenBank
Sichuan Xichang	KF279407.1	China: Sichuan, Xichang	GenBank
SJYEIIm10	HM120847.1	China: Yunnan, Eryuan	GenBank
HNYY06	KU196343.1	-	GenBank
HNCD04	KU196331.1	-	GenBank
AHTL10	KU196317.1	-	GenBank
AHTL05	KU196312.1	-	GenBank
AHGC07	KU196305.1	-	GenBank
PH0010	KU196388.1	-	GenBank
PH0001	KU196379.1	-	GenBank
IN0008	KU196355.1	-	GenBank
HBSS05	KU196322.1	-	GenBank
SJASNM6	JQ781208.1	Philippines: Asuncion	GenBank
SJLEYF5	JQ781209.1	Philippines: Leyte	GenBank
SJSXM24	HM120846.1	China: Sichuan, Tianquan	GenBank
SJSORM5	JQ781214.1	Philippines: Sorsogon	GenBank
SJSORF5	JQ781213.1	Philippines: Sorsogon	GenBank
SJMINM6	JQ781212.1	Philippines: Mindoro	GenBank
SJMINF5	JQ781211.1	Philippines: Mindoro	GenBank
SJASNF5	JQ781206.1	Philippines: Asuncion	GenBank
SjMDMU	LC733209.1	Japan	GenBank
SjFDMU	LC733208.1	Japan	GenBank
JAP	EF635954.1	Philippines: Leyte	[5]
SJYYM2	JQ781215.1	Japan: Yamanashi	GenBank
Taiwan Changhua	KF279410	Taiwan, Changhua	GenBank

Supplementary Table 3. Bacterial strains and constructs used in this study.

Strain or plasmid	Relevant features	Reference(s)
**Strain**		
NEB10-beta	Δ(*ara -leu*) 7697 *araD139 fhuA* Δ*lacX74 galK16 galE15 e14*-ϕ*80*d*lacZ*Δ*M15 recA1 relA1endA1 nupG rpsL* (StrR) *rph spoT1* Δ(*mrr-hsdRMS-mcrBC*); cloning strain	New England Biolabs
**Plasmid**		
pCR-Blunt-II-TOPO	Cloning vector for PCR products; KanR, ZeoR	Invitrogen
pAJW333	NEB10-beta pCR-Blunt-II-TOPO-SJ-*cox*1; 446 base pair *cox*1 gene sequence fragment taken from GenBank entry EU325878.1, synthesised by IDT and cloned into pCR-II-TOPO; KanR, NeoR	This study

Supplementary Table 4. Δ fluorescence (a.u.) per hour measurements for *S. japonicum* probe sets 2, 3, 4 and 5 against corresponding target sequences from related species

Probe and ssDNA target combination	Δ fluorescence (a.u.) per hour	Mean Δ fluorescence (a.u.) per hour	Standard error of the Mean
Probe 2 SJ_WT9	30052, 29668, 31266, 22709, 21569, 22704, 25861, 25346, 26775	26217	1176
Probe 2 Smek_Smal_WT9	-95, -123, -101, -175, -159, -83, -84, -87, -85	-110.2	11.58
Probe 2 SM_WT9	-137, -59, -152, -213, -116, -83, -93, -55, -63	-107.9	17.46
Probe 2 SR_WT9	-97, -123, -143, -168, -143, -276, -71, -59, -95	-130.6	21.72
Probe 2 SH_WT9	-99, -173, -124, -849, -158, -102, -111, -107, -97	-202.2	81.34
Probe 2 SB_WT9	-70, -108, -175, -148, -210, -133, -114, -109, -75	-126.9	15.13
Probe 2 SG_WT9	-143, -173, -184, -146, -94, -159, -58, -20, -40	-113	20.45
Probe 2 SC_WT9a	-95, -87, -126, -172, -200, -108, -53, -84, -9	-103.8	19.27
Probe 2 SC_WT9b	-103, -137, -141, -159, -159, -129, -37, -81, -100	-116.2	13.35
Probe 2 No DNA	-83, -111, -139, -119, -107, -180, -12, -75, -45	-96.78	16.68
Probe 3 SJ_WT10	17474, 17229, 18072, 13271, 13952, 14372, 14969, 14649, 14877	15429	571.2
Probe 3 Smek_WT10	-138, -160, -135, -186, -154, -151, -96, -39, -23	-120.2	18.70
Probe 3 Smal_WT10	5242, 5171, 5723, 5286, 5366, 5641, 6905, 6869, 6896	5900	254.6
Probe 3 SM_WT10	123, 621, 615, 464, 444, 433, 581, 524, 545	483.3	50.77
Probe 3 SR_WT10	1157, 1222, 1185, 1228, 1080, 1023, 1228, 1097, 1191	1157	24.65
Probe 3 SH_SC_WT10	-190, -134, -77, -140, -163, -119, -70, -4, -91	-109.8	18.65
Probe 3 SB_WT10a	-112, -93, -119, -203, -149, -111, -97, -63, -78	-113.9	13.85
Probe 3 SB_WT10b	-211, -145, -63, -150, -167, -113, -54, -27, -15	-105	22.77
Probe 3 SG_WT10	-173, -132, -121, -133, -164, -172, -96, -47, -76	-123.8	14.66
Probe 3 No DNA	-133, -167, -110, -101, -146, -158, -83, -91, -76	-118.3	11.23
Probe 4 SJ_WT11	29789, 30117, 32290, 29589, 30443, 30860, 23575, 22512, 23468	28071	1253
Probe 4 Smek_WT11	1320, 1365, 1403, 1267, 1333, 1325, 864, 774, 817	1163	87.36
Probe 4 Smal_WT11	-118, -175, -190, -109, -43, -149, -119, -91, -17	-112.3	18.91
Probe 4 SM_WT11	-209, -116, -93, -63, -95, -90, -74, -57, -48	-93.89	16.06
Probe 4 SR_WT11	-161, -248, -185, -84, -68, -52, -137, -85, -111	-125.7	21.15
Probe 4 SH_WT11	-191, -192, -146, -51, -44, -60, -116, -84, -77	-106.8	19.23
Probe 4 SB_WT11	-202, -162, -201, -73, -72, -85, -117, -111, -70	-121.4	18.01
Probe 4 SG_WT11	-200, -178, -151, -92, -61, -44, -122, -59, -58	-107.2	19.32
Probe 4 SC_WT11	-179, -190, -227, -79, -80, -49, -100, -60, -63	-114.1	22.08
Probe 4 No DNA	-173, -150, -169, -19, -42, -76, -75, -22, -110	-92.89	20.22
Probe 5 SJ_WT12	38931, 39002, 38982, 40116, 41309, 41011, 32083, 33970, 32993	37600	1191
Probe 5 Smek_WT12	-185, -152, -189, -104, -44, -141, -87, -71, -84	-117.4	17.16
Probe 5 Smal_WT12	-177, -127, -192, -125, -67, -113, -86, -106, -121	-123.8	13.22

Probe 5 SM_WT12	-166, -194, -168, -40, -130, -55, -98, -89, -124	-118.2	17.50
Probe 5 SR_WT12	-160, -173, -185, -142, -89, -4, -15, 2, -113	-97.67	25
Probe 5 SH_WT12	-119, -175, -165, 5, -30, -25, -58, -100, -61	-80.89	21
Probe 5 SB_SG_SC_WT12	-165, -178, -130, -77, -106, -36, -92, -21, -39	-93.78	18.84
Probe 5 No DNA	-157, -187, -177, -44, -31, -29, -74, -49, -86	-92.67	21.30

Data is shown in Fig. 1 of the main manuscript. Δ fluorescence (a.u.) per hour was calculated using raw fluorescence values between 20 and 80 minutes of the plate reader assay. Measurements were obtained using a BMG CLARIOstar plate reader (Ex. 440-15 nm/ Em. 510-20 nm, 1500 gain). Mean and Standard error of the mean calculated using GraphPad Prism 10.4.1.

Supplementary Table 5. Δ fluorescence (a.u.) per hour measurements for *S. japonicum* probe sets 2, 3, 4 and 5 against ssDNA derived from plasmids containing the 446-base *S. japonicum*-specific *cox*1 target region

Probe and ssDNA target combination	Δ fluorescence (a.u.) per hour	Mean Δ fluorescence (a.u.) per hour	Standard error of the Mean
probe2_SJ1	55433, 56645, 62301	58126	2116
probe2_SJ2	61212, 62606, 65333	63050	1210
probe2_SJ3	62594, 65502, 65250	64449	930.2
probe2 No DNA	-421, -464, -479	-454.7	17.38
probe3_SJ1	1451, 1668, 1373	1497	88.25
probe3_SJ2	946, 1263, 1381	1197	129.9
probe3_SJ3	1634, 1319, 848	1267	228.4
probe3 No DNA	-744, -408, -535	-562.3	97.95
probe4_SJ1	744, 850, 297	630.3	169.5
probe4_SJ2	254, 10, 1105	456.3	331.9
probe4_SJ3	83, 241, 938	420.7	262.7
probe4 No DNA	-616, -670, -360	-548.7	95.61
probe5_SJ1	22705, 22901, 22359	22655	158.4
probe5_SJ2	21986, 21167, 21794	21649	247.3
probe5_SJ3	19456, 19341, 19296	19364	47.64
probe5 No DNA	-758, -547, -618	-641.0	61.99

Data is shown in Fig. 2 of the main manuscript. Δ fluorescence (a.u.) per hour was calculated using raw fluorescence values between 20 and 80 minutes of the plate reader assay. Measurements were obtained using a BMG CLARIOstar plate reader (Ex. 440-15 nm/ Em. 510-20 nm, 2000 gain). Mean and Standard error of the mean calculated using GraphPad Prism 10.4.1.

Supplementary Table 6. Sequences of *cox*1 amplified from either *S. japonicum* or *S. mansoni* cercarial gDNA

Sample number	Notes	Sequence
SJ1	*S. japonicum*, Tongqiao<br>OZ203289	TTTAGCTACTTCTGGTGTTGGTGTGGATTACTTAATGTTCTCTT<br>TACATCTTGCTGGTGTATCTAGTTTGATTGGTTCTATAAATTTT<br>ATTACTACTATAATGTTGCGTCTAAGGTCATGTTCTTCAGTTAT<br>TAGATGATCTTATTTATTTACTTCGGTGTTGTTATTGTTATCGT<br>TGCCGGTTCTTGCTGCAGGTATAACTATGTTGTTGTTTGATCGT<br>AAATTTGGTACTGCTTTTTTTGAGCCAGCAGGTGGTGGTGATC<br>CTGTGTTATTTCAACATTTATTTGGTTTTTGGTCACCCAGAA<br>GTATATGTTTTGATATTGCCTGGATTTGGTATAGTAAGTCATA<br>TATGTATGTCTTTAAGTAATAATAATTCTTCGTTTGGATATTAT<br>GGGTTAGTTTGTGCCATGGGTTCTATTGTG
SJ2	*S. japonicum*, Tongqiao<br>OZ203290	TTTAGCTACTTCTGGTGTTGGTGTGGATTACTTAATGTTCTCTT<br>TACATCTTGCTGGTGTATCTAGTTTGATTGGTTCTATAAATTTT<br>ATTACTACTATAATGTTGCGTCTAAGGTCATGTTCTTCAGTTAT<br>TAGATGATCTTATTTATTTACTTCGGTGTTGTTATTGTTATCGT<br>TGCCGGTTCTTGCTGCAGGTATAACTATGTTGTTGTTTGATCGT<br>AAATTTGGTACTGCTTTTTTTGAGCCAGCAGGTGGTGGTGATC<br>CTGTGTTATTTCAACATTTATTTGGTTTTTGGTCACCCAGAA<br>GTATATGTTTTGATATTGCCTGGATTTGGTATAGTAAGTCATA<br>TATGTATGTCTTTAAGTAATAATAATTCTTCGTTTGGATATTAT<br>GGGTTAGTTTGTGCCATGGGTTCTATTGTGTGTTTGGGGAG
SJ3	*S. japonicum*, Chenbi<br>OZ203291	TTAGCTACTTCTGGTGTTGGTGTGGATTACTTAATGTTCTCTTT<br>ACATCTTGCTGGTGTATCTAGTTTGATTGGTTCTATAAATTTTA<br>TTACTACTATAATGTTGCGTCTAAGGTCATGTTCTTCAGTTATT<br>AGATGATCTTATTTATTTACTTCGGTGTTGTTATTGTTATCGTT<br>GCCGGTTCTTGCTGCAGGTATAACTATGTTGTTGTTTGATCGT<br>AAATTTGGTACTGCTTTTTTTGAGCCAGCAGGTGGTGGTGATC<br>CTGTGTTATTTCAACATTTATTTGGTTTTTGGTCACCCAGAA<br>GTATATGTTTTGATATTGCCTGGATTTGGTATAGTAAGTCATA<br>TATGTATGTCTTTAAGTAATAATAATTCTTCGTTTGGATATTAT<br>GGGTTAGTTTGTGCCATGGGTTCTATTGTGTGTT
SJ4	*S. japonicum*, Chenbi<br>OZ203292	TTCTTTAGCTACTTCTGGTGTTGGTGTGGATTACTTAATGTTCT<br>CTTTACATCTTGCTGGTGTATCTAGTTTGATTGGTTCTATAAAT<br>TTTATTACTACTATAATGTTGCGTCTAAGGTCATGTTCTTCAGT<br>TATTAGATGATCTTATTTATTTACTTCGGTGTTGTTATTGTTAT<br>CGTTGCCGGTTCTTGCTGCAGGTATAACTATGTTGTTGTTTGAT<br>CGTAAATTTGGTACTGCTTTTTTTGAGCCAGCAGGTGGTGGTG<br>ATCCTGTGTTATTTCAACATTTATTTGGTTTTTGGTCACCCA<br>GAAGTATATGTTTTGATATTGCCTGGATTTGGTATAGTAAGTC<br>ATATATGTATGTCTTTAAGTAATAATAATTCTTCGTTTGGATAT<br>TATGGGTTAGTTTGTGCCATGGGTTCTATTGTGTGTTTGGGGA<br>G
SJ5	*S. japonicum*, Chenbi<br>OZ203293	CTTTGTCTTCTTTAGCTACTTCTGGTGTTGGTGTAGATTACTTA<br>ATGTTCTCTTTACATCTTGCTGGTGTATCTAGTTTGATTGGTTC<br>TATAAATTTTATTACTACTATAATGTTGCGTCTAAGGTCATGTT<br>CTTCAGTTATTAGATGATCTTATTTATTTACTTCGGTGTTGTTA<br>CTGTTATCGTTGCCGGTTCTTGCTGCAGGTATAACTATGTTGTT<br>GTTTGATCGTAAATTTGGTACTGCTTTTTTTGAGCCAGCAGGT<br>GGTGGTGATCCTGTGTTATTTCAACATTTATTTGGTTTTTGG<br>TCACCCAGAAGTATATGTTTTGATATTGCCTGGATTTGGTATA<br>GTAAGTCATATATGTATGTCTTTAAGTAATAATAATTCTTCGTT<br>TGGATATTATGGGTTAGTTTGTGCTATGGGTTCTATTGTGTGT
SJ6	*S. japonicum*, Chenbi<br>OZ203294	CTTCTTTAGCTACTTCTGGTGTTGGTGTAGATTACTTAATGTTC<br>TCTTTACATCTTGCTGGTGTATCTAGTTTGATTGGTTCTATAAA<br>TTTTATTACTACTATAATGTTGCGTCTAAGGTCATGTTCTTCAG<br>TTATTAGATGATCTTATTTATTTACTTCGGTGTTGTTACTGTTA<br>TCGTTGCCGGTTCTTGCTGCAGGTATAACTATGTTGTTGTTTGA<br>TCGTAAATTTGGTACTGCTTTTTTTGAGCCAGCAGGTGGTGGT<br>GATCCTGTGTTATTTCAACATTTATTTGGTTTTTGGTCACCC<br>AGAAGTATATGTTTTGATATTGCCTGGATTTGGTATAGTAAGT<br>CATATATGTATGTCTTTAAGTAATAATAATTCTTCGTTTGGATA<br>TTATGGGTTAGTTTGTGCTATGGGTTCTATTGTGTGTTT
SJ7	*S. japonicum*, Jianhong<br>OZ203295	TTTAGCTACTTCTGGTGTTGGTGTGGATTATTTAATGTTCTCTT<br>TACATCTTGCTGGTGTATCTAGTTTGATTGGTTCTATAAATTTT<br>ATTACTACTATAATGTTGCGTCTAAGGTCATGTTCTTCAGTTAT<br>TAGATGATCTTATTTATTTACTTCGGTGTTGTTACTGTTATCGT<br>TGCCGGTTCTTGCTGCAGGTATAACTATGTTGTTGTTTGATCGT<br>AAATTTGGTACTGCTTTTTTTGAGCCAGCAGGTGGTGGTGATC<br>CTGTGTTATTTCAACATTTATTTGGTTTTTGGTCACCCAGAA<br>GTATATGTTTTGATATTGCCTGGATTTGGTATAGTAAGTCATA

		TATGTATGTCTTTAAGTAATAATAATTCTTCGTTTGGATATTAT<br>GGGTTAGTTTGTGCTATGGGTTCTATTGTG
SJ8	*S. japonicum*, Jianhong<br>OZ203296	TTCTTTAGCTACTTCTGGTGTTGGTGTGGATTATTTAATGTTCT<br>CTTTACATCTTGCTGGTGTATCTAGTTTGATTGGTTCTATAAAT<br>TTTATTACTACTATAATGTTGCGTCTAAGGTCATGTTCTTCAGT<br>TATTAGATGATCTTATTTATTTACTTCGGTGTTGTTACTGTTAT<br>CGTTGCCGGTTCTTGCTGCAGGTATAACTATGTTGTTGTTTGAT<br>CGTAAATTTGGTACTGCTTTTTTTGAGCCAGCAGGTGGTGGTG<br>ATCCTGTGTTATTTCAACATTTATTTTGGTTTTTTGGTCACCCA<br>GAAGTATATGTTTTGATATTGCCTGGATTTGGTATAGTAAGTC<br>ATATATGTATGTCTTTAAGTAATAATAATTCTTCGTTTGGATAT<br>TATGGGTTAGTTTGTGCTATGGGTTCTATTGTGTGTTT
SJ9	*S. japonicum*, Jianhong<br>OZ203297	TTCTTTAGCTACTTCTGGTGTTGGTGTGGATTATTTAATGTTCT<br>CTTTACATCTTGCTGGTGTATCTAGTTTGATTGGTTCTATAAAT<br>TTTATTACTACTATAATGTTGCGTCTAAGGTCATGTTCTTCAGT<br>TATTAGATGATCTTATTTATTTACTTCGGTGTTGTTACTGTTAT<br>CGTTGCCGGTTCTTGCTGCAGGTATAACTATGTTGTTGTTTGAT<br>CGTAAATTTGGTACTGCTTTTTTTGAGCCAGCAGGTGGTGGTG<br>ATCCTGTGTTATTTCAACATTTATTTTGGTTTTTTGGTCACCCA<br>GAAGTATATGTTTTGATATTGCCTGGATTTGGTATAGTAAGTC<br>ATATATGTATGTCTTTAAGTAATAATAATTCTTCGTTTGGATAT<br>TATGGGTTAGTTTGTGCTATGGGTTCTATTGTGTGTTT
SJ10	*S. japonicum*, Shundi<br>OZ203298	CCTTTGTCTTCTTTAGCTACTTCTGGTGTTGGTGTGGATTACTT<br>AATGTTCTCTTTACATCTTGCTGGTGTATCTAGTTTGATTGGTT<br>CTATAAATTTTATTACTACTATAATGTTGCGTCTAAGGTCATGT<br>TCTTCAGTTATTAGATGATCTTATTTATTTACTTCGGTGTTGTT<br>ATTGTTATCATTGCCGGTTCTTGCTGCAGGTATAACTATGTTGC<br>TGTTTGATCGTAAATTTGGTACTGCTTTTTTTGAGCCAGCAGGT<br>GGTGGTGATCCTGTGTTATTTCAACATTTATTTTGGTTTTTTGG<br>TCACCCAGAAGTATATGTTTTGATATTGCCTGGATTTGGTATA<br>GTAAGTCATATATGTATGTCTTTAAGTAATAATAATTCTTCGTT<br>CGGATATTATGGGTTAGTTTGTGCTATGGGTTCTATTGTGTGTT<br>T
SJ11	*S. japonicum*, Shundi<br>OZ203299	CCTTTGTCTTCTTTAGCTACTTCTGGTGTTGGTGTGGATTACTT<br>AATGTTCTCTTTACATCTTGCTGGTGTATCTAGTTTGATTGGTT<br>CTATAAATTTTATTACTACTATAATGTTGCGTCTAAGGTCATGT<br>TCTTCAGTTATTAGATGATCTTATTTATTTACTTCGGTGTTGTT<br>ATTGTTATCGTTGCCGGTTCTTGCTGCAGGTATAACTATGTTGT<br>TGTTTGATCGTAAATTTGGTACTGCTTTTTTTGAGCCAGCAGGT<br>GGTGGTGATCCTGTGTTATTTCAACATTTATTTTGGTTTTTTGG<br>TCACCCAGAAGTATATGTTTTGATATTGCCTGGATTTGGTATA<br>GTAAGTCATATATGTATGTCTTTAAGTAATAATAATTCTTCGTT<br>TGGATATTATGGGTTAGTTTGTGCCATGGGTTCTATTGTGTGTT<br>TGGGGAG
SJ12	*S. japonicum*, Shundi<br>OZ203300	TTCTTTAGCTACTTCTGGTGTTGGTGTGGATTACTTAATGTTCT<br>CTTTACATCTTGCTGGTGTATCTAGTTTGATTGGTTCTATAAAT<br>TTTATTACTACTATAATGTTGCGTCTAAGGTCATGTTCTTCAGT<br>TATTAGATGATCTTATTTATTTACTTCGGTGTTGTTATTGTTAT<br>CGTTGCCGGTTCTTGCTGCAGGTATAACTATGTTGTTGTTTGAT<br>CGTAAATTTGGTACTGCTTTTTTTGAGCCAGCAGGTGGTGGTG<br>ATCCTGTGTTATTTCAACATTTATTTTGGTTTTTTGGTCACCCA<br>GAAGTATATGTTTTGATATTGCCTGGATTTGGTATAGTAAGTC<br>ATATATGTATGTCTTTAAGTAATAATAATTCTTCGTTTGGATAT<br>TATGGGTTAGTTTGTGCCATGGGTTCTATTGTGTGTTT
SJ13	*S. japonicum*, Shundi<br>OZ203301	TTCTTTAGCTACTTCTGGTGTTGGTGTGGATTACTTAATGTTCT<br>CTTTACATCTTGCTGGTGTATCTAGTTTGATTGGTTCTATAAAT<br>TTTATTACTACTATAATGTTGCGTCTAAGGTCATGTTCTTCAGT<br>TATTAGATGATCTTATTTATTTACTTCGGTGTTATTATTGTTAT<br>CGTTGCCGGTTCTTGCTGCAGGTATAACTATGTTGTTGTTTGAT<br>CGTAAATTTGGTACTGCTTTTTTTGAGCCAGCAGGTGGTGGTG<br>ATCCTGTGTTATTTCAACATTTATTTTGGTTTTTTGGTCACCCA<br>GAAGTATATGTTTTGATATTGCCTGGATTTGGTATAGTAAGTC<br>ATATATGTATGTCTTTAAGTAATAATAATTCTTCGTTTGGATAT<br>TATGGGTTAGTTTGTGCTATGGGTTCTATTGTGTGTTT
SJ14	*S. japonicum*, Shundi<br>OZ203302	TTCTTTAGCTACTTCTGGTGTTGGTGTGGATTACTTAATGTTCT<br>CTTTACATCTTGCTGGTGTATCTAGTTTGATTGGTTCTATAAAT<br>TTTATTACTACTATAATGTTGCGTCTAAGGTCATGTTCTTCAGT<br>TATTAGATGATCTTATTTATTTACTTCGGTGTTATTATTGTTAT<br>CGTTGCCGGTTCTTGCTGCAGGTATAACTATGTTGTTGTTTGAT<br>CGTAAATTTGGTACTGCTTTTTTTGAGCCAGCAGGTGGTGGTG<br>ATCCTGTGTTATTTCAACATTTATTTTGGTTTTTTGGTCACCCA<br>GAAGTATATGTTTTGATATTGCCTGGATTTGGTATAGTAAGTC<br>ATATATGTATGTCTTTAAGTAATAATAATTCTTCGTTTGGATAT<br>TATGGGTTAGTTTGTGCTATGGGTTCTATTGTGTGTTTGGGGA<br>G

SJ15	*S. japonicum*, Shundi<br>OZ203303	CTTCTTTAGCTACTTCTGGTGTTGGTGTAGATTACTTAATGTTC<br>TCTTTACATCTTGCTGGTGTATCTAGTTTGATTGGTTCTATAAA<br>TTTTATTACTACTATAATGTTGCGTCTAAGGTCATGTTCTTCAG<br>TTATTAGATGATCTTATTTATTTACTTCGGTGTTGTTATTGTTA<br>TCGTTGCCGGTTCTTGCTGCAGGTATAACTATGTTGTTGTTTGA<br>TCGTAAATTTGGTACTGCTTTTTTTGAGCCAGCAGGTGGTGGT<br>GATCCTGTGTTATTTCAACATTTATTTTGGTTTTTTGGTCACCC<br>AGAAGTATATGTTTTGATATTGCCTGGATTTGGTATAGTAAGT<br>CATATATGTATGTCTTTAAGTAATAATAATTCTTCGTTTGGATA<br>TTATGGGTTGGTTTGTGCTATGGGTTCTATTGTGTGTTT
SJ16	*S. japonicum*, Shundi<br>OZ203304	CTTCTTTAGCTACTTCTGGTGTTGGTGTAGATTACTTAATGTTC<br>TCTTTACATCTTGCTGGTGTATCTAGTTTGATTGGTTCTATAAA<br>TTTTATTACTACTATAATGTTGCGTCTAAGGTCATGTTCTTCAG<br>TTATTAGATGATCTTATTTATTTACTTCGGTGTTGTTATTGTTA<br>TCGTTGCCGGTTCTTGCTGCAGGTATAACTATGTTGTTGTTTGA<br>TCGTAAATTTGGTACTGCTTTTTTTGAGCCAGCAGGTGGTGGT<br>GATCCTGTGTTATTTCAACATTTATTTTGGTTTTTTGGTCACCC<br>AGAAGTATATGTTTTGATATTGCCTGGATTTGGTATAGTAAGT<br>CATATATGTATGTCTTTAAGTAATAATAATTCTTCGTTTGGATA<br>TTATGGGTTGGTTTGTGCTATGGGTTCTATTGTGTGTTT
SM1	*S. mansoni*, laboratory-maintained strain	ATTTGAGAGGGGTCTGGTTTTGGTGTAGATTATTTAATGTTTTC<br>TCTTCATTTGGCAGGGGTTTCAAGTCTAATTGGATCTGTCAAT<br>TTCATTTCTACGATTTTTAGTCGTTTAAGATTCAAATGTTCGAT<br>AATAGTATGGGCTTATCTATTTACGTCTGTTTTATTATTGCTTT<br>CGTTACCTGTGTTAGCCAGAGGAATAACGATGTTATTATTTGA<br>TCGTAAATTTGGAACTGCTTTTTTTGAGCCGTCAGGCGGTGGC<br>GATCCTATTTTGTTTCAGCATTTATTTTGGTTTTTTGGTCATCC<br>AGAGGTTTATGTTTTGATCCTTCCGGGTTTTGGTATAGTTAGG<br>CATATCTGTATGAGTCTAAGGAATAAAGATTCGTCGTTTGGTT<br>ATTATGGATTGATTTGCGCTATGGCTTCTATAGTATGC
SM2	*S. mansoni*, laboratory-maintained strain	CAATTTGAGAGGGGTCTGGTTTTGGTGTAGATTATTTAATGTT<br>TTCTCTTCATTTGGCAGGGGTTTCAAGTCTAATTGGATCTGTCA<br>ATTTCATTTCTACGATTTTTAGTCGTTTAAGATTCAAATGTTCG<br>ATAATAGTATGGGCTTATCTATTTACGTCTGTTTTATTATTGCT<br>TTCGTTACCTGTGTTAGCCAGAGGAATAACGATGTTATTATTT<br>GATCGTAAATTTGGAACTGCTTTTTTTGAGCCGTCAGGCGGTG<br>GCGATCCTATTTTGTTTCAGCATTTATTTTGGTTTTTTGGTCAT<br>CCAGAGGTTTATGTTTTGATCCTTCCGGGTTTTGGTATAGTTAG<br>GCATATCTGTATGAGTCTAAGGAATAAAGATTCGTCGTTTGGT<br>TATTATGGATTGATTTGCGCTATGGCTTCTATAGTATGC
SM3	*S. mansoni*, laboratory-maintained strain	CAATTTGAGAGGGGTCTGGTTTTGGTGTAGATTATTTAATGTT<br>TTCTCTTCATTTGGCAGGGGTTTCAAGTCTAATTGGATCTGTCA<br>ATTTCATTTCTACGATTTTTAGTCGTTTAAGATTCAAATGTTCG<br>ATAATAGTATGGGCTTATCTATTTACGTCTGTTTTATTATTGCT<br>TTCGTTACCTGTGTTAGCCAGAGGAATAACGATGTTATTATTT<br>GATCGTAAATTTGGAACTGCTTTTTTTGAGCCGTCAGGCGGTG<br>GCGATCCTATTTTGTTTCAGCATTTATTTTGGTTTTTTGGTCAT<br>CCAGAGGTTTATGTTTTGATCCTTCCGGGTTTTGGTATAGTTAG<br>GCATATCTGTATGAGTCTAAGGAATAAAGATTCGTCGTTTGGT<br>TATTATGGATTGATTTGCGCTATGGCTTCTATAGTATGC

Bases highlighted in red indicate sequence variation from that of *S. japonicum* GenBank entry EU325878.1.

Supplementary Table 7. Δ fluorescence (a.u.) per hour measurements for *S. japonicum*-specific probe 2 against ssDNA derived from cercariae samples SJ1-SJ16 and SM1-SM3

ssDNA target	Δ fluorescence (a.u.) per hour	Mean Δ fluorescence (a.u.) per hour	Standard error of the Mean
SJ1	51420, 50232, 50596	50749	351.4
SJ2	50058, 50885, 50015	50319	283.1
SJ3	48452, 48307, 49962	48907	529.2
SJ4	49204, 49896, 50492	49864	372.2
SJ5	28840, 29739, 30003	29527	352
SJ6	36377, 35920, 34892	35730	439.1
SJ7	42633, 42475, 43810	42973	421.1
SJ8	41886, 42872, 44156	42971	657.2
SJ9	29800, 30121, 30191	30037	120.4
SJ10	44306, 43837, 43464	43869	243.6
SJ11	44718, 44307, 45907	44977	479.7
SJ12	42736, 45011, 45246	44331	800.4
SJ13	35053, 34802, 35441	35099	185.9
SJ14	36850, 35929, 36735	36505	289.7
SJ15	52032, 53062, 49416	51503	1085
SJ16	44342, 45292, 44363	44666	313.2
SM1	-635, -465, -517	-539	50.29
SM2	-686, -231, -494	-470.3	131.9
SM3	-239, -718, -294	-417	151.3
No DNA	-702, -662, -673	-679	11.93

Data is shown in Fig. 3 of the main manuscript. Δ fluorescence (a.u.) per hour was calculated using raw fluorescence values between 20 and 80 minutes of the plate reader assay. Measurements were obtained using a BMG CLARIOstar plate reader (Ex. 440-15 nm/ Em. 510-20 nm, 2000 gain). Mean and Standard error of the mean calculated using GraphPad Prism 10.4.1.

References

1. Edgar, R. C. MUSCLE: multiple sequence alignment with high accuracy and high throughput. *Nucleic Acids Res* **32**, (2004).

2. Ying, Z.-M. *et al.* Spinach-based fluorescent light-up biosensors for multiplexed and label-free detection of microRNAs. *Chemical Communications* **54**, (2018).

3. Webb, A. J. *et al.* Specific Nucleic AcId Ligation for the detection of Schistosomes: SNAILS. *PLoS Negl Trop Dis* **16**, e0010632 (2022).

4. Young, N. D. *et al.* Exploring molecular variation in Schistosoma japonicum in China. *Sci Rep* **5**, 17345 (2015).

5. Attwood, S. W., Fatih, F. A. & Upatham, E. S. DNA-Sequence Variation Among Schistosoma mekongi Populations and Related Taxa; Phylogeography and the Current Distribution of Asian Schistosomiasis. *PLoS Negl Trop Dis* **2**, (2008).
