# Supplementary material for: Species-specific detection of Schistosoma japonicum using the ‘SNAILS’ DNA-based biosensor
Source: Commun Biol. 2025 Aug 30;8:1321. doi: 10.1038/s42003-025-08773-7 (PMC12398544; doi:10.1038/s42003-025-08773-7)
Supplement: Supplementary file 2 — Description of Additional Supplementary Files [file 42003_2025_8773_MOESM2_ESM.docx]

Description of Additional Supplementary Files

**File name:** Supplementary Data

**Description:** Excel spreadsheet containing the source data behind the graphs in the paper.
